# Role of neuroinflammation in neurodegeneration development

**DOI:** 10.1038/s41392-023-01486-5

**Published:** 2023-07-12

**Authors:** Weifeng Zhang, Dan Xiao, Qinwen Mao, Haibin Xia

**Affiliations:** 1 Laboratory of Gene Therapy, Department of Biochemistry, College of Life Sciences, Shaanxi Normal University, 199 South Chang’an Road, Xi’an, 710062 P.R. China; 2 The State Laboratory of Cancer Biology, Department of Biochemistry and Molecular Biology, Air Force Medical University, No. 169 Changle West Road, Xi’an, 710032 P.R. China; 3 Department of Burns and Cutaneous Surgery, Xijing Hospital, Air Force Medical University, No. 169 Changle West Road, Xi’an, 710032 China; 4 Department of Pathology, University of Utah, Huntsman Cancer Institute, 2000 Circle of Hope Drive, Salt Lake City, UT 84112 USA

**Keywords:** Diseases of the nervous system, Neurodevelopmental disorders, Immunological disorders

## Abstract

Studies in neurodegenerative diseases, including Alzheimer’s disease, Parkinson’s disease and Amyotrophic lateral sclerosis, Huntington’s disease, and so on, have suggested that inflammation is not only a result of neurodegeneration but also a crucial player in this process. Protein aggregates which are very common pathological phenomenon in neurodegeneration can induce neuroinflammation which further aggravates protein aggregation and neurodegeneration. Actually, inflammation even happens earlier than protein aggregation. Neuroinflammation induced by genetic variations in CNS cells or by peripheral immune cells may induce protein deposition in some susceptible population. Numerous signaling pathways and a range of CNS cells have been suggested to be involved in the pathogenesis of neurodegeneration, although they are still far from being completely understood. Due to the limited success of traditional treatment methods, blocking or enhancing inflammatory signaling pathways involved in neurodegeneration are considered to be promising strategies for the therapy of neurodegenerative diseases, and many of them have got exciting results in animal models or clinical trials. Some of them, although very few, have been approved by FDA for clinical usage. Here we comprehensively review the factors affecting neuroinflammation and the major inflammatory signaling pathways involved in the pathogenicity of neurodegenerative diseases, including Alzheimer’s disease, Parkinson’s disease, and Amyotrophic lateral sclerosis. We also summarize the current strategies, both in animal models and in the clinic, for the treatment of neurodegenerative diseases.

## Introduction

Neurodegeneration corresponds to any pathological condition primarily affecting neurons. In clinical practice, neurodegenerative diseases represent a large group of neurological disorders, with various clinical and pathological characteristics affecting specific subsets of neurons in specific regions of the central nervous system (CNS). Classical neurodegenerative diseases include Alzheimer’s disease (AD), Parkinson’s disease (PD), Amyotrophic lateral sclerosis (ALS), frontotemporal dementia (FTD) and Huntington’s disease (HD). Although these diseases have different pathogenetic mechanisms, such as different protein aggregates and genetic variations, they all share the hallmark of chronic neuroinflammation.^1^

More than 200 years ago, James Parkinson first described the neurodegenerative disease now known as PD.^2^ This was the first medical description of PD, although ancient Chinese medical sources from approximately 425 BC and traditional Indian texts from approximately 1000 BC described conditions that resemble PD.^3^ After that, ALS and AD were described in 1869 and 1906, respectively. At first, the molecular mechanisms underlying neurodegenerative diseases were largely focused on gross anatomical changes, including protein aggregation (e.g., amyloid beta (Aβ), neurofibrillary tangles (NFTs), TAR DNA binding protein 43 (TDP-43)) and neuronal damage. In 1975, the presence of immune-related proteins in the senile plaques of AD patients was first reported.^4^ In the 1980s, microglia activation has been identified as a feature of neurodegenerative diseases.^5–8^ But at that time, inflammation was only considered as a standby phenomenon induced by the other AD pathologies, one that increased the severity of disease, but was not the fundamental cause. Beginning in the 1990s, several studies found that individuals who were long-term nonsteroidal anti-inflammatory drugs (NSAIDs) users had as much as a 50% reduction in the risk of developing AD.^9^ At the same time, numerous papers elucidating the origin, nature, and toxicity of microglia, as well as the relationship between microglia and neurodegenerative diseases.^10–13^ These research efforts led to a reevaluation of the role of inflammation in neurodegeneration, and there has been increasing recognition that neuroinflammation may play a central role in the development of neurodegenerative diseases.

At present, there is mounting evidence of the importance of immune responses in neurodegeneration, particularly growing immune-related genetic mutations have been suggested to be risk factors for neurodegeneration. More and more underlying molecular mechanisms have been revealed, which provides compelling evidence for developing therapeutic strategies that regulate neuroinflammation to prevent CNS pathologies. In this review, we mainly focus on the relationship between neuroinflammation and the pathology of neurodegenerative diseases, the vital inflammatory signaling pathways involved neurodegeneration, and the therapeutic strategies targeting inflammatory signaling pathways.

## Probable inducers of inflammation in neurodegeneration

**Fig. 1:**
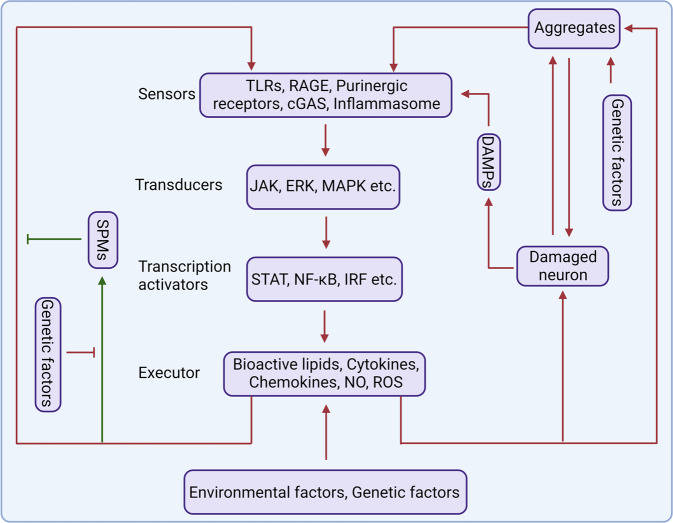
The role of inflammation in neurodegeneration. Inflammatory receptors on the surface of immune cells, especially glial cells, act as sensors to detect abnormality in the human body. Stimulation of the sensors by DAMPs or PAMPs, such as protein aggregates, virus, bacteria, leads to activation of signal transducers which then activate transcription activators. Subsequently, activated transcription factors induce the secretion of inflammatory mediators which further amplify inflammation. Generally, activated glial cells should kill the dangers and then induce an inflammation resolution process to clear the DAMPs or PAMPs and stop inflammatory response. However, owing to some reasons, activated immune cells fail to resolve inflammation and generate chronic inflammation which cause neuronal toxicity and enhance protein aggregation. Protein aggregates and DAMPs released from damaged neurons further amplify neuroinflammation and aggravate disease. Some protein aggregates, such as TDP-43 and α-synuclein, may even invade mitochondria which can induce death of neuron directly

Neurodegenerative diseases are featured by many shared and diverse pathological and clinical characteristic, including the selective susceptibility of brain regions and the aggregation of different proteins. In addition to the neuropathological and clinical characteristics of neurodegenerative diseases, they also exhibit persistent and chronic inflammation.^14^ Inflammation was previously thought to be a result of protein aggregations in the CNS; however, increasing evidence demonstrate that immune signaling may not just be a consequence of protein aggregation in the brain, but that it actually causes the buildup of aggregates at the earliest stages of the disease process.^15–17^ The immune system plays crucial roles in the maintenance of tissue homeostasis, removal of pathogens, and injury recovery.^18,19^ In most cases, the immune response is beneficial and self-limited, and it is resolved once the tissue injury has been repaired or the infection has been eliminated. But in some cases, due to the failure to clear an inflammatory stimulus, normal resolution mechanisms become overwhelmed, resulting in chronic inflammation that may lead to the release of neurotoxic factors and exacerbated disease. A persistent stimulus may be caused by endogenous factors (e.g., protein aggregates),^14,20^ environmental factors (e.g., systematic infection, gut commensal dysbiosis, aging, diet),^21–23^ and genetic susceptibility (e.g., progranulin (PGRN) mutations, apolipoprotein E4 (APOE4) mutations).^24,25^ In addition, a group of bioactive lipids, named specialized pro-resolving lipid mediators (SPMs), can be induced during inflammation and promote the resolution of inflammation, which are important for the recovery of tissue from inflammation. Failure of resolution owing to the reduced production of SPMs is another reason that leads to chronic inflammatory diseases^26^ (Fig. 1).

### Endogenous factors

#### Alzheimer’s disease

**Fig. 2:**
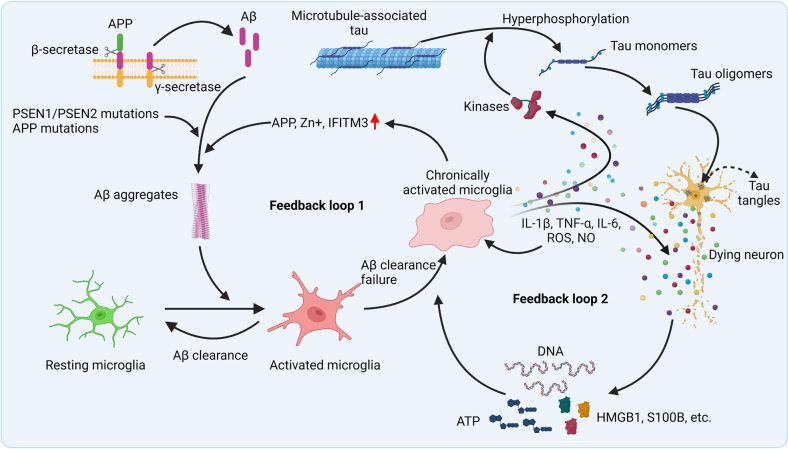
Inflammation in AD. In the earliest stages of AD, the formation of Aβ occurs due to abnormal cleavage of APP by β- and γ-secretases. Aβ monomers are intrinsically disordered and have a propensity to oligomerize and aggregate into Aβ plaques, which can be promoted by genetic mutations in APP or PSEN1/PSEN2 genes. The Aβ aggregates activates microglia, causing them to clear Aβ via phagocytosis and proteolysis. Yet when that clearance fails, microglia become chronically activated, which further enhances the aggregation of Aβ by improving the expression of APP and IFITM3 and increasing the release of irons, such as Zn^+^. This process forms a positive feedback loop, which leads to persistent, chronic inflammation. Chronically activated microglia also release proinflammatory cytokines and toxic products, including ROS and nitric oxide (NO), which amplify the immune response and lead to neurotoxicity. DAMPs released from dying neurons, including ATP, HMGB1, S100B, DNA, etc., also amplify inflammation and lead to the second positive feedback loop. Inflammatory cytokines activate kinases, leading to hyperphosphorylation of tau, the dissociation of tau monomers from microtubules, and the subsequent formation of tau tangles in the cytosol of neurons. Thus, inflammation acts as a link between the aggregation of Aβ and the accumulation of tau tangles

AD, which was initially described by Alois Alzheimer in 1907, is the most common neurodegenerative disease. More than 10% of people older than 65 and 30% of people older than 85 are affected by AD,^27^ and the numbers are growing very fast. The pathology of AD is characterized by extracellular amyloid plaques and intracellular NFTs, which are surrounded by immune cells, especially microglia; the clinical manifestation of AD is characterized by progressive cognitive impairment.^14^ The main component of the amyloid plaques is amyloid beta (Aβ), which is generated by improper cleavage of the amyloid precursor protein (APP), and the NFTs are composed of hyperphosphorylated microtubule-binding protein tau. The accumulation of Aβ and the deposition of NFTs have been considered two core pathogenic causes of AD, although why and how that accumulation and deposition occur has yet to be clarified.^28^ Aβ aggregation begins in the preclinical phase of AD. Patients may exhibit Aβ plaque pathology for more than a decade before any clinical diagnosis of AD,^29,30^ while tau pathology usually occurs downstream from the deposition of Aβ plaques.^31^ It is thought that deposited Aβ can act as a damage-associated molecular pattern (DAMP) and bind to receptors like toll-like receptors (TLRs), receptor for advanced glycation end products (RAGE), and nucleotide-binding oligomerization domain-like receptors (NLRs). This binding activates the surrounding microglia, which release numerous cytokines and chemokines and recruit more glial cells to the Aβ locus.^32^ Activated microglia and astrocytes can phagocytize Aβ through multiple receptors to protect neurons,^33,34^ but failure to clear the aggregates leads to persistent chronic inflammation and the release of a variety of proinflammatory and toxic products, including cytokines, chemokines, reactive oxygen species (ROS) and nitric oxide (NO), which amplify immune responses and lead to neurotoxicity.^35^ Other CNS cells, such as neurons, oligodendrocytes, vascular endothelial cells and pericytes, may also contribute to the maintenance of the inflammatory microenvironment.^36^ Activated microglia may be involved in the generation of Aβ plaque by increasing the secretion of Aβ fragments; inducing the expression of interferon-induced transmembrane protein 3 (IFITM3), a γ-secretase modulatory protein; or releasing agents such as iron, which enhances the aggregation of soluble β-amyloid.^37–39^ The activation of glial cells may provide a link between the initial Aβ aggregation and the later development of tau aggregates,^40^ since activation of microglia precedes tau aggregation^41^ and promotes tau hyperphosphorylation which subsequently leads to the formation of NFTs.^42^ The accumulation of tau tangles in the nervous fiber further leads to the loss of neuronal function, and ultimately apoptosis^43^ and immune cell activation^44^ (Fig. 2). NFTs have also been found to be spatially correlated with neuroinflammation in clinical samples from AD patients.^45^ Meng et al. further demonstrated that the hyperphosphorylated tau can disrupt membrane bilayers and activate human macrophages through TLR4.^46^ Recently, Welikovitch et al. found that soluble and oligomeric amyloid protein-burdened neurons exhibited a unique inflammatory profile. This neuron-specific inflammatory response may even precede insoluble Aβ plaque and tau tangle formation, implicating the intraneuronal accumulation of Aβ as a very early event during AD development and suggesting that there is a significant immunological component to AD pathogenesis.^47^

In addition to aggregates, tissue injury also induces the extracellular release of DAMPs, such as mitochondrial DNA (mtDNA), high mobility group box 1 (HMGB1), S100 proteins, chromogranin A, adenosine 5′-triphosphate (ATP), and uric acid, from damaged brain cells. These DAMPs trigger neuroinflammation by activating pattern recognition receptors (PRRs), including TLRs, RAGE, NLRs, P2Y receptors among others, which are expressed either on the surface of cerebral myeloid cells or intracellularly.^48^

In summary, it has been clear that the pathogenic protein aggregation and neuroinflammation exhibit a mutual-promotion pattern to aggravate neurodegeneration. But it is still not clear which one is the initiator. The “amyloid cascade hypothesis” suggests that certain elements in the human induce Aβ deposition which further leads to inflammation and neurodegeneration. Protein aggregations, like Aβ and tau, have long been considered as ideal targets for AD therapy. However, people with the genetic mutations that can directly lead to protein aggregations (e.g., APP, PSEN1, PSEN2) only account for a very small part of AD patients. For most of the AD patients, other inducements should exist. In addition, therapies aimed at clearing the aggregates have got limited success to suppress the progression of AD so far. These results indicate that protein aggregation may not be the initiator, or not be the only initiator, other elements common to AD patients may be crucial for the development and progression of AD. Mounting evidence suggests that inflammation is a crucial player in this process.

#### Parkinson’s disease

PD, the second most common neurodegenerative disease, is pathologically characterized by the presence of Lewy bodies, which is composed of aggregated α-synuclein, and the loss of dopaminergic neurons in the substantia nigra.^49^ Dopaminergic neurons, which located in the substantia nigra, send their axons to the striatum. These projections are particularly important for motor functions, so PD is clinically characterized by a range of motor symptoms.^50^ The onset of disease usually occurs decades before the first symptom appears. Analysis of postmortem tissues of PD patients has shown that about 30% of dopamine neuronal cell bodies in the substantia nigra and 50% of dopamine axon terminals in the dorsal putamen have been lost at the time of diagnosis.^51^ At 4 years after the initial diagnosis, almost all of the dopamine axon terminals are lost, while the majority of the neuronal cell bodies are lost 5 years after diagnosis.

**Fig. 3:**
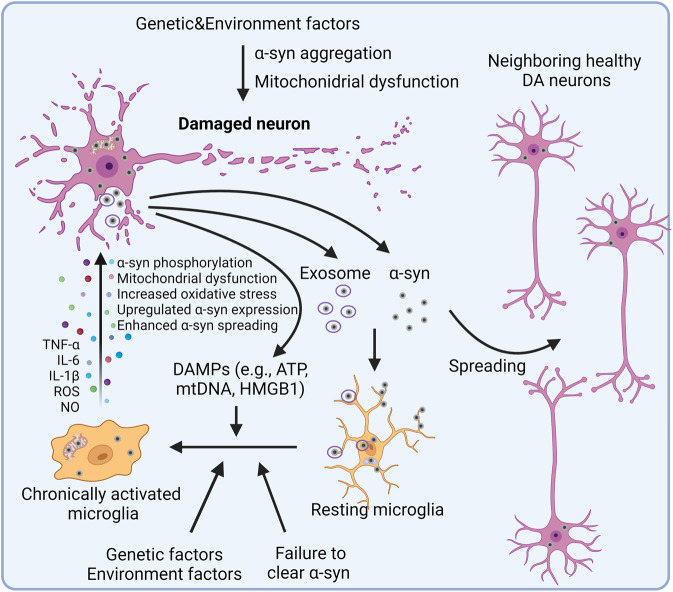
Inflammation in PD. Lewy bodies, mainly composed of α-synuclein aggregates, are one of the pathological features of PD. It is generally accepted that some genetic and environment factors lead to the aggregation of α-synuclein. Excessive α-synuclein in neuron is transported into the mitochondria, leading to the mitochondrial dysfunction that is central to the progression of PD. Mutations in mitochondrial-associated proteins like LRRK2, PINK1, PARK7, and PRKN, which are found in familial cases of PD, also induce mitochondrial dysfunction and lead to neurotoxicity. Excessive aggregation of α-synuclein or failure to clear it from the cell will result in its release either directly from the neuron or through the exosome, which activates microglia and amplifies neurotoxicity by spreading α-synuclein to the neighboring healthy DA neurons. DAMPs released from dying neurons further enhance the activation of microglia. In addition, genetic and environment factors also promote the activation of microglia and other immune cells. Activated microglia further exacerbate disease by enhancing α-synuclein pathogenicity, increasing oxidative stress, and promoting mitochondrial dysfunction

In 1998, McGeer et al. found HLA-HR^+^ reactive microglia in the postmortem tissue of PD patients. This was the first report of the involvement of inflammation in PD.^6^ At present, there are lots of evidence showing that inflammation may be an early event and play a crucial role during the development of PD. Both genetic mutations and post-translational modifications (e.g., α-synuclein at the S129 site) likely induced by environmental factors can lead to conformational changes and the formation of insoluble plaques.^51^ But α-synuclein aggregations are not the first cause of neuronal death in PD, as neuronal loss is observed prior to Lewy pathology.^52^ Although in vivo injection of misfolded α-synuclein induces toxicity in neurons, the involvement of inflammation cannot be excluded as an underlying mechanism.^53^ Actually, the direct function of α-synuclein aggregations to neuron is still controversial. In some in vitro experiments, misfolded α-synuclein showed no toxicity to neurons unless microglia were present, indicating that microglia activation may be crucial for the toxicity of α-synuclein aggregates to neurons.^54,55^ But other in vitro studies revealed that oligomeric α-synuclein induced mitochondrial dysfunction and neuronal death through multiple mechanisms.^56,57^ Indeed, numerous studies in PD animal models have revealed that microglia were activated prior to the death of dopamine neurons.^58–60^ In animal models of PD and in PD patients, misfolded α-synuclein was found to be released from injured neurons into the extracellular fluid.^51,61,62^ This extracellular α-synuclein can further activate microglia or astrocytes through binding to the specific surface receptors (e.g., TLRs, FcγR) or to intracellular receptors (e.g., NLRP3).^49,63,64^ Activated glial cells release a band of cytokines and chemokines that induce the death of neurons, leading to the release of new DAMPs (e.g., ATP).^65^ In a healthy brain, most α-synuclein is unphosphorylated; however, approximately 90% of α-synuclein in the Lewy bodies of patients with PD is phosphorylated at Ser129, which is presumed to be of pathological significance.^66^ Proinflammatory cytokines released from activated microglia may induce phosphorylation of α-synuclein at Ser129 by activating protein kinase R (PKR).^67^ But Ghanem et al. found that Ser129 phosphorylation occurred after the initial α-synuclein aggregation and inhibited further aggregation, indicating Ser129 phosphorylation has a potential protective role.^68^ Activated glial cells may also exacerbate disease by promoting the prion-like spreading of aggregates and enhancing α-synuclein expression^69–71^ (Fig. 3). In contrast, Scheiblich et al. reported that α-synuclein fibril-burdened microglia transfer α-synuclein to neighboring naïve microglia through the formation of F-actin-dependent intercellular connections, thus promoting the degradation of α-synuclein.^72^ In addition, microglia can also attach to the cell membrane of astrocytes, attracting and clearing intracellular protein deposits, such as α-synuclein and Aβ, from the astrocytes.^73^ A possible explanation is that α-synuclein is transferred from α-synuclein burdened glial cells to neighboring naïve microglia to attract more glial cells involving in the degradation of α-synuclein, but excessive α-synuclein phagocytosis may induce chronic activation of microglia and provide the seed for microglia-to-neuron transmission.^70^ Recently, numerous evidences suggest that adaptive immune system plays important role in approximately 40% PD. A high number of T cells response to α-synuclein were found in the brain of PD patients and mouse model. Variation in genetic factors or environmental factors may promote the generation of α-synuclein responsive T cells. Microglia activation by α-synuclein aggregates or other factors can increase the expression of MHC I expression on neurons, enhance the presentation of α-synuclein antigens by neurons, which were subsequently killed by α-synuclein reactive T cells.^74–76^ These findings suggest PD to be an auto-immune disease, which is similar to multiple sclerosis.

Together, α-synuclein is an important player in the progression of PD, but should not be the initiator of PD, since the presence of α-synuclein pathology is much later than microglia activation and the loss of dopaminergic neurons. Actually, growing evidences support immune response, including innate immune response and adaptive immune response, to be a driver, rather than a consequence, of neuronal death. For successful therapy of PD, it is important to intervene the progression of disease before loss of dopaminergic neurons. Developing new methods for the diagnosis of PD as early as possible is crucial prognosis of disease. Since immune dysregulation is an early phenomenon during the development of PD, it should be a promising target for PD therapy.

#### Amyotrophic lateral sclerosis

**Fig. 4:**
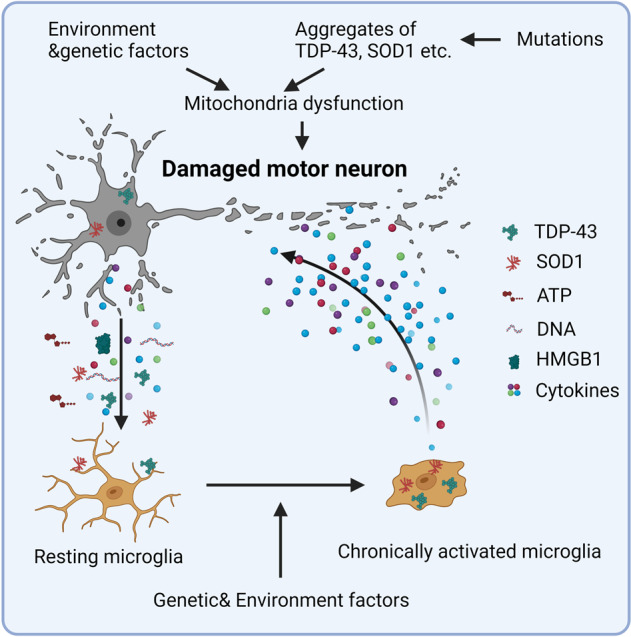
Inflammation in ALS. TDP-43 or SOD1 forms aggregates in the cytoplasm due to genetic and/or environmental factors, causing deleterious effects to neuron. SOD1 aggregation induces oxidative stress, while TDP-43 aggregates invade mitochondria and release mtDNA into the cytoplasm, inducing inflammation through the cGAS-STING pathway. Some mitochondria related genetic variants directly lead to dysfunction of mitochondria and oxidative stress in motor neurons. Proinflammatory cytokines and DAMPs released from damaged motor neurons activate microglia and other immune cells, leading to a persistent inflammatory attack on the motor neurons. Genetic and environment factors also promote the activation of microglia and other immune cells directly, benefiting the development of ALS

ALS, first described by Charcot in 1869, is a neurodegenerative disease characterized by the progressive degeneration of upper and lower motor neurons in the brain and spinal cord. ALS is always fatal, with an average life expectancy of 2–5 years after diagnosis.^77^ The progression of ALS toward the fatal outcome is extremely rapid—faster than any other neurodegenerative disease. Most ALS is sporadic, while about 5–10% is familial. More than 20 gene variations have been found in familial ALS, with chromosome 9 open reading frame 72 (C9orf72, 40%) and Cu/Zn superoxide dismutase 1 (SOD1, 20%) as the most frequent genetic causes of familial ALS.^78^ In 2006, hyperphosphorylated and ubiquitinated TDP-43 (encoded by *TARDBP*) cytoplasmic inclusions were identified as the most common (at about 97%) pathological hallmark of ALS.^79^ Multiple factors may contribute to the development and progression of ALS, including aggregates (formed by TDP-43, SOD1, FUS, etc.) in the nucleus, cytoplasm, or extracellular matrix which induce cellular damage and neuronal dysfunction, loss of function mutations (e.g., mutations in SOD1, C9orf72, and TDP-43) and environmental factors.^80^ TDP-43 aggregates, the primary pathogenic factor of ALS, can invade mitochondria, release mtDNA into the cytosol, and induce inflammation in neurons through activating cGAS/STING pathway.^81^ Aggregation of SOD1, a major cytoplasmic antioxidant enzyme and widely studied pathogenic factor for ALS, induces oxidative stress and leads to mitochondrial dysfunction.^82^ Protein aggregates released into the extracellular space together with DAMPs released from damaged neurons induce microglia activation and proinflammatory cytokines releasing.^20,83,84^ Persistent inflammation damages neurons directly and exacerbates ALS^78,85^ (Fig. 4). Moreover, patients with autoimmune diseases showed a slightly increased frequency of developing ALS.^86^ A genome-wide association study also demonstrated a specific genetic correlation between ALS and autoimmune diseases.^87^ Therefore, inflammation should have close relationship with the development of ALS.

In conclusion, the endogenous pathologic protein aggregations can induce neuroinflammation, which further enhances protein aggregation and promotes neurodegeneration. Actually, inflammation seems to play a crucial role in the development and progression of neurodegenerative diseases. Mounting evidence suggests that common environment risk factors of neurodegenerative diseases can trigger an inflammatory response, initiating and exacerbating the progression of disease. In addition, many of the genetic risk factors of neurodegenerative diseases are immune-related genes. These data indicate that inflammation probably plays a central role in the initiation and progression of neurodegeneration.

### Environmental factors

#### Traumatic brain injury (TBI)

TBI induces a widespread neuroinflammatory response that can promote recovery if controlled for a defined time period. But if the traumatized tissue is not adequately repaired, it may generate a stable, low-grade irritation, which will induce chronic inflammation and provoke continuous damage to the surrounding tissue. Indeed, increased microglia activation has been found many years after injury in some TBI patients. Chronic neuroinflammation ultimately induces neurological impairment and neurodegeneration.^88^ Interestingly, diffuse Aβ plaques can be detected in the brain of 30% of individuals that acutely die from TBI.^89^ In addition, TBI was found to induce APP accumulation in injured axons.^88^ TBI has also been found to be a risk factor for PD and ALS.^90,91^

#### Systemic inflammation

It has been reported that individuals with higher levels of inflammatory proteins in the blood during midlife (decades before the typical age of dementia symptom onset) are at increased risk for developing neurodegenerative disease.^92^ Similarly, elevations in inflammatory proteins during midlife have been associated with smaller brain volumes and abnormal white matter microstructural integrity 20+ years later, during late-life.^93^ Together, these findings suggest that systemic inflammation from the peripheral immune system, such as that caused by infection or gut microbiota perturbation, occurring decades before the typical age of dementia onset, may promote the progression of neurodegenerative processes.^94^ The CNS has traditionally been known as an immune-privileged region; however, increasing evidences show that peripheral immune cells reside at the borders of brain, including the choroid plexus and the meninges, surveil the CNS and transport the antigens from the CNS to the deep cervical lymph nodes via the drainage of CSF.^95,96^ Actually, peripheral immune cells, even including the self-reactive T cells, have been found to be important for the homeostasis of CNS.^97^ But imbalanced immune response in the CNS leads to neuropathology. Multiple clinical and experimental studies have demonstrated that peripheral inflammatory molecules induced by acute systemic viral or bacterial infections can affect brain function through neural and humoral pathways.^94,98,99^ In addition, activated peripheral immune cells can also infiltrate the CNS and activate microglia; together, both microglia and the peripheral immune cells can induce inflammation and Aβ deposition in the CNS.^100^ For example, herpes simplex virus (HSV) infection continually triggers the immune system through frequent cycles of latency and reactivation. However, our immune system cannot completely eradicate the virus. As a result, HSV infection-induced persistent inflammation leads to Aβ peptide overproduction and aggregation.^101^ COVID-19, a pandemic affecting more than five hundred million people around the world in the past 3 years, has been found to induce acute parkinsonism. Scientists are worrying that it may also elevated long-term risk of PD.^102^ In recent years, the relationship between gut microbiota and neurodegenerative disease has been reported by many studies. Gut microbiota imbalance induces intestinal inflammation and further leads to systemic inflammation through the release of immune-stimulating substances and metabolites into blood circulation.^103^ Moreover, gut bacteria can also affect CNS inflammation by binding to specific receptors, such as the proinflammatory tachykinin/neurokinin receptors, in the vagus nerve fiber.^104^

#### Chronic inflammation

From an epidemiological perspective, chronic diseases like diabetes, obesity, atherosclerosis, and depression, which are associated with chronic inflammation, increase the risk of developing neurodegeneration. Obesity was found to be associated with a state of chronic, low-grade systemic inflammation.^105^ In obese patients, dysregulated lipid metabolism leads to increased levels of free fatty acids in circulation, which can bind to PRRs on immune cells and induce the release of proinflammatory cytokines, including interleukin-6 (IL-6), tumor necrosis factor α (TNF-α), adipokines, and monocyte chemoattractant protein-1 (MCP-1),^106^ which also disrupt mitochondrial function and stimulate the production of ROS.^107^ Consequently, these inflammation mediators can disrupt the blood-brain barrier (BBB), enter the brain, induce persistent chronic neuroinflammation, and subsequently result in neurodegeneration.^106^ Dietary patterns greatly affect the levels and composition of circulating lipids in the plasma (e.g., the ratio between saturated and unsaturated fatty acids), which also affects the activation of immune cells in the CNS.^108,109^ For example, mice fed to a high-fat diet experienced lipid droplet formation in their astrocytes, which subsequently signaled microglia to augment the inflammatory response.^110^ Diabetes is another disease that increases the risk of neurodegeneration, and higher blood glucose levels are considered a major contributing factor.^111^ Bahniwal et al. reported that astrocytes exposed to high glucose in vitro secreted more IL-6 and Interleukin 8.^112^ Moreover, microglial response to lipopolysaccharide (LPS), a known inflammation inducer, is enhanced under high glucose conditions.^113^ These results indicate that high glucose may affect the development of neurodegenerative diseases by enhancing the activation of microglia and astrocytes, but the precise mechanisms need further investigation. Depression and dementia often occur together, according to the clinical and epidemiological data. Actually, loneliness and other elements of depression may precede and worsen the progression of dementia. This comorbidity may at least partly induced by inflammation.^114^

#### Aging

Aging is a major environmental factor for many neurodegenerative diseases and is accompanied by low-grade systemic inflammation, which is termed “inflammaging”.^49^ Over the course of an individual’s life, microglia and astrocytes are activated again and again, causing oxidative stress, free radical damage, and mtDNA to accumulate, which makes them more likely to have a “primed” phenotype and morphology. They show an elevated baseline inflammatory state, a more vigorous proinflammatory response after receiving a stimulation, and a loss in their ability to maintain homeostasis.^115^ At the systemic level, peripheral inflammaging leads to low-level production of inflammatory mediators in the circulation, including IL-6, TNF-α, and C-reactive protein (CRP), which also contribute to the inflammatory environment in the CNS.^36,49,116^ Moreover, numerous studies have shown an increased BBB permeability in aged mice, which facilitates the infiltration of peripheral immune cells into the CNS.^117,118^ However, more investigations are needed to delineate the exact molecular mechanisms of inflammaging. Recently, Minhas et al. reported an interesting finding. They found that the bioenergetics of myeloid cells from aging mice are suppressed by increased lipid messenger prostaglandin E2 (PGE2), which leads to an energy-deficient state and drives maladaptive proinflammatory responses. Inhibiting peripheral myeloid PGE2 signaling is sufficient to restore cognition in aged mice. This study demonstrates that dysregulated immune functions can be reversed by reprogramming glucose metabolism and that glucose metabolism in myeloid cells could be a target for therapy.^119^

#### Circadian rhythms

Disruption of the sleep/wake cycle is a shared symptoms among different neurodegenerative diseases.^120^ An important function of circadian rhythms is to modulate the time-specific activities of the immune system. It has been reported that circadian locomotor output cycles protein kaput (CLOCK), cryptochrome (CRY) and brain and muscle ARNT-like protein 1 (BMAL1) can regulate the secretion of cytokines and chemokines through many immune mediator genes.^121^ In vivo BMAL1 deletion was found to induce activation of glial cells and degeneration of presynaptic axonal terminals.^122^ Further studies suggested that BMAL1 may affect glial cells activation and neurodegeneration through REV-ERBα, which is a nuclear receptor as well as a circadian clock component that can be induced by BMAL1.^123–125^ In line with the close relationship between circadian rhythms and neurodegeneration, melatonin, a natural hormone produced by the brain to regulate night and day cycles, has been demonstrated to be useful for treating neurodegenerative diseases.^126^

#### Exercise

Exercise training has been shown to be a powerful tool in combating neuroinflammation and cognitive dysfunction in patients with AD, PD, ALS, HD, frontotemporal dementia (FTD), MS. Many studies have demonstrated that exercise inhibited neuroinflammation, ameliorated oxidative stress, and reduced neuron loss.^127^ Reversal of epigenetic clock is reported to be an underlying mechanism.^128^ Recently, De Miguel et al. found that an infusion of the plasma from a voluntarily running mouse into a sedentary mouse could reduce brain inflammation in the latter. Plasma proteomic analysis revealed that physical exercise increased the level of complement cascade inhibitors in the plasma of voluntarily running mice. Clusterin, one of the complement cascade inhibitors, can bind to the receptor on brain endothelial cells and reduce the expression of inflammatory genes in an AD mouse model.^129^ Exercise may also influence the permeability of the BBB,^130^ thus reducing the infiltration of activated peripheral immune cells.

#### Cerebrovascular impairment

Cerebrovascular impairment has been suggested to be associated with neurodegenerative diseases, including AD, PD, and ALS. Cerebrovascular impairment causes chronic cerebral hypoperfusion, ischemia, or hypoxia, all of which lead to oxidative stress and induce microvascular inflammation. Cerebrovascular impairment also increases the infiltration of peripheral immune cells into the CNS, which then cooperate with CNS resident immune cells to induces neuroinflammation.^131^ BBB and the blood–cerebrospinal fluid barrier (BCSFB) are two important specialized borders between the blood and CNS parenchymal tissue or cerebrospinal fluid (CSF), protecting CNS against harmful substances in the blood and providing an immune privilege in the CNS.^132^ In recent years, mounting evidence suggested that vascular pathologic mechanisms are involved in the development of AD.^133^ For example, vascular pathologic signs have been found in 79.9% of AD patients.^134^ Recently, an important study published by Yang et al. further highlighted the crucial role of brain vascular cells in AD development. Although recent studies strongly implicate microglia as the major AD GWAS genes-expressing cells, with the expanded survey of brain cell types, Yang et al. found that actually at least 30 of the top 45 AD GWAS genes are highly expressed in human cerebrovasculature cells, suggesting thorough vascular and perivascular involvement in AD.^135^

In mouse model of AD and AD patients, BBB benefits the clearance of Aβ aggregates through the efflux of amyloid from the brain by dedicated transporters, such as LRP1 (lipoprotein receptor-related protein-1) and P-glycoprotein,^136,137^ while vascular risk factors induced microvascular inflammation disrupts BBB and reduces the efflux of Aβ from the brain, leading to Aβ deposits onto the vasculature which further impairs vascular function.^138^ So vascular risk factors induce a positive feedback loop that leads to chronic impairment of the function of BBB and enhanced aggregation of Aβ. BBB impairments were also found to trigger TGF-β signaling in astrocytes and impair cognition in rodent.^139^ Actually, there are increasing evidence showing that BBB breakdown is among the early markers in AD development.^140,141^ Recently, using LPS stimulated mouse model, Zhao et al. found that maternal immune activation (MIA) induced disruption of BBB formation at the fetal stage led to chronic brain inflammation persisting across the offspring life span, suggesting that gestational MIA disruption of BBB formation could be an etiological contributor to neuropsychiatric disorders.^142^ These results indicate that BBB impairments not only act as a participant of neuroinflammation and AD, but also can act as the initiator. The role of two major BBB constituent cells, including endothelial cells and pericytes, in neuroinflammation and AD development has also been discussed in the following parts.

The choroid plexuses (CPs), located within the brain ventricles, are composed of a tight polarized epithelium responsible for CSF secretion, which surrounds a loose connective core containing highly fenestrated blood vessels and cells of the lymphoid lineage.^143^ These epithelial cells joined by tight junctions form the BCSFB, which rigorously regulates the exchange of substances between the CSF and blood. In addition to secrete CSF, CP also acts as an important neuro-immunological interface that integrates signals from the CNS parenchyma and circulating immune cells. It also functions as an on-alert gate for selective authorizing the entry of inflammation-resolving leukocytes to the inflamed CNS region.^26^ In addition, CP also displays important function in the removal of neurotoxic compounds (e.g., Aβ plaques) from the CSF.^144^

Numerous studies have revealed AD associated BCSFB disruption induced by proinflammatory cytokines or matrix metalloproteinase (MMP) expressed by CP epithelial cells. BCSFB disruption impaired the selectivity of it, leading to increased infiltration of cytokines and leukocytes into the CSF which then activated glial cells in the CNS.^144,145^ BCSFB acts as a gateway for inflammation-resolving leukocyte entry into the CSF during immune responses through the constitutively expressed adhesion molecules and chemokines in CP epithelial cells.^26^ Shechter et al. found that the homing of proinflammatory macrophage and resolving macrophages derived from circulating monocytes to traumatized spinal cord was distinctly regulated. The proinflammatory macrophages were recruited to the injured region in the CNS through the adjacent leptomeninges in a CCL2 dependent manner, while the resolving macrophages were preferentially recruited to the CNS through CP.^146^ IFN-γ was verified as the crucial player in the expression of resolving macrophages attracting factors by the choroid plexus epithelium. In spinal cord injury mouse model, knockout of IFN-γ receptor reduced the infiltration of T cells and monocytes to the CSF, impaired the resolution of inflammation and attenuated recovery.^147^ In addition, it has been found that the level of IFN-γ was reduced at the CP of aging brain,^148^ indicating an attenuated ability to resolve inflammation in aging brain. This phenomenon was also found in neurodegenerative diseases, including ALS and AD.^149,150^ Using a 5xFAD AD mouse model, found that the secretion of IFN-γ from CP may be suppressed by Treg cells. Depletion of Treg cells restored the secretion of IFN-γ and the function of CP.^150^ In addition, the ratio between type I interferon and IFN-γ also affect the function of CP in AD mouse model.^151^ Treatment of 5xFAD AD mouse with PD-1 antibody increased splenocyte frequencies of IFN-γ producing CD4+ T cells, this systematically enhanced IFN-γ secretion in the peripheral also enhanced the homing of monocyte derived macrophages to the CNS through CP, leading to clearance of Aβ plaques and improved cognitive performance.^152^ Immunostaining of the in vitro cell culture and the choroid plexus from mice revealed that TNF-α and IFN-γ reciprocally control the expression of their receptors in the choroid plexus, through which way they synergistically activate the choroid plexus epithelium to express trafficking molecules.^147^ Excessive nitric oxide (NO) production is a common phenomenon found in neurodegenerative diseases, including AD. Exposure of CP epithelial cells to NO impaired TNF-α induced nuclear translocation of NFκB/p65, which is responsible for the inhibitory effect of NO on the expression of leukocyte trafficking determinants. In 5xFAD AD mouse model, systemic administration of an NO scavenger attenuated NFκB/p65 suppression and restored the gateway activity of CP.^153^ In conclusion, signals from CNS and peripheral affect the gateway activity of CP, reduce the infiltration of inflammation resolving cells, thus exacerbate disease.

Together, inflammation dysregulation in the peripheral or CNS can initiate or exacerbate brain pathology, while cerebrovascular impairment benefits the inflammatory signal communication between the peripheral and CNS, subsequently amplifying inflammation in the CNS. Immune-related environmental risk factors sensitize the onset and progression of neurodegenerative diseases. The immune response to environmental risk factors may also be affected by immune-related genetic variations. The recent advances on anti-inflammatory therapy further confirmed the importance of inflammation on neurodegeneration. In China, a phase 3 trial of GV971, a sodium oligomannate that reported to remodel the gut microbiota and attenuate inflammation in AD mice,^154^ improved cognitive functions in AD, and has been approved for the treatment of patients with mild to moderate AD in 2019. A phase 3 trial in the US/Canada is ongoing. Moreover, loss of function variation in TREM1 was indicated to be responsible for the defective clearance of *P. gingivalis* and gingipains from the brain, resulting in chronic, low-level infection and neuroinflammation in susceptible individuals.^155^ A phase 2/3 trial of COR388 (a gingipain inhibitor) was ongoing to determine whether it can improve cognition of patients with mild to moderate AD.

### Genetic factors

#### Alzheimer’s disease

Although largely sporadic (~90–95%), AD has a very strong genetic component.^156^ Since the 1930s, it has been known that the early onset AD which represents rare form of AD (up to 5% of all people with AD) is fully genetically determined. But it was until 1980s that three genes, including APP, PSEN1, and PSEN2, were found to be responsible for early onset AD.^157^ These rare mutations cause the aggregation of amyloid proteins, leading to the progression of early onset AD. This hypothesis greatly changed our understanding of AD. For late onset “sporadic” AD (occur after 65 years of age), disease relate variants have been found in a much larger number of genes, but their contribution to disease risk is generally lower.^156^ Until 2020, more than 40 genes/loci have been linked to the AD risk,^158^ the number of entire AD risk genes should be even larger. Although the lower contribution of gene variants to ‘sporadic’ AD, these genetic knowledges have greatly improved our perception of the pathogenesis of AD. Among the risk genes, mutations were observed especially in genes related to the function and activation of immune cells, particularly microglia,^158,159^ clearly implicating the crucial role of inflammation in neurodegeneration.

##### APOE

APOE is a lipoprotein that responsible for the transport of lipids through binding to its receptors on the cell surface. It was discovered in 1993 and found to be the most predominant genetic risk factor for late onset AD.^160^ Notably, APOE plays important role on the modulating of immune responses and has been found to have anti-inflammatory effects in multiple mouse models.^161^ In a study using primary microglia and astrocytes isolated from knockout mice, APOE knockout was found to significantly enhance the secretion of proinflammatory cytokines from microglia and reduce the release of anti-inflammatory cytokines from microglia and astrocytes.^162^ In normal human brains, APOE is mainly derived from astrocytes,^35,163^ while its expression by microglia is induced by ageing as well as amyloid and tau pathology.^35,164,165^ There are three isoforms of APOE gene in humans, respectively, APOE2, APOE3, APOE4. Compared with APOE3, the APOE4 isoform increases AD risk and theAPOE2 isoform reduces AD risk.^166^ A single amino acid difference between APOE3 and APOE4 leads to the changing of protein conformation which affects the binding of APOE with its receptors, lipids, and Aβ.^167^ Carrying one APOE4 allele increases the risk of AD by 3–4 times, and carrying two alleles increases the risk by 9–15 times.^168^ APOE4 isoform may affects the development and progression of AD through multiple different pathways in which inflammatory regulation plays crucial role.^166^

Lipid accumulation is a prevalent phenomenon presented in both AD patients and AD mouse model.^169^ Given that brain is an organ rich in lipid, lipid homeostasis is extremely important for normal brain function. As a key lipid transport protein, APOE is important for the lipid homeostasis in the brain. Thus, carrying APOE4 isoform may impair the lipid transport and disrupt the lipid homeostasis in the brain, inducing chronic inflammation and subsequently lead to neurodegeneration.^170^ The brain is also a highly energy-demanding organ, dysfunction of brain glucose metabolism is another risk factor for AD development.^171^ Mounting in vitro and in vivo evidences have demonstrated that APOE4 can inhibit insulin signaling and decrease glucose usage in the brain through multiple mechanisms,^172–174^ this might be another way APOE4 induces neuroinflammation and promotes the development of AD. Recently, higher level of pro-inflammatory eicosanoid lipidome was found in APOE4 carrying older persons with AD compared with non-carriers, indicating that APOE4 may affect the molecules involved in eicosanoid metabolism.^175^ In addition, APOE4 was found to reduce the phagocytosis ability of glial cells, enhance microglia proinflammatory activation by Aβ plaques or tau aggregates and promote neurodegeneration in the mouse models.^176,177^ Although the underlying molecular mechanism is still far from clear, some clues have been found. Study using APOE4 target replacement mice revealed that APOE4 can increase the expression of miRNA146a,^178^ which was found to be highly expressed in AD, reducing the expression of its target protein complement factor H, an important repressor of the inflammatory response of the brain.^179^ Using astrocyte differentiated from human induced pluripotent stem cells, Arnaud et al. found that APOE4 decreased the expression of Transgelin 3 in astrocytes, ultimately led to the activation of astrocytes through NF-kB pathway.^180^ But there are also some conflicting results. Triggering receptor expressed on myeloid cell 2 (TREM2) is an immune regulatory receptor mainly expressed in myeloid cells. Recently, APOE was found to be a ligand of TREM2 and bind to TREM2 with a high affinity.^181^ Krasemann et al. found that APOE can bind with the intracellular domain of TREM2 and switch microglia from a homeostatic phenotype to neurodegenerative phenotype in multiple neurodegenerative models including AD, indicating APOE-TREM2 pathway as a proinflammatory signaling.^182^ Except microglia and astrocytes, APOE was also found to regulate the activation of pericytes. As we discussed above, neurovascular impairment is also an important risk factor for AD. Through analyzing the samples from AD patient and AD mouse model, APOE was also found to affect the integrity of BBB through regulating the activation and apoptosis of pericytes,^183^ which plays crucial role in BBB integrity.^184^ Transgenic expression of human APOE4 in the mice led to uncontrolled expression of proinflammatory cyclophilin A (CypA) in pericytes, inducing the activation of NF-kB and matrix metalloproteinase 9 (MMP9), which in turn results in BBB breakdown.^183^ APOE4 even induces cognitive decline independent of Aβ or tau pathology probably through CypA-MMP9 pathway.^185^ In line with the vital role of APOE4 mediate inflammation in the progression of AD, recent epidemiological data suggest that APOE4 carriers response better to NSAIDs treatment.^186^

##### TREM2

Whole-genome analysis led to the identification of relatively rare mutations in TREM2 gene that are associated with a high AD risk.^187^ Given the TREM2 is exclusively expressed in immune cells,^188^ and involved in the activation of immune cells by stimulating phagocytosis and reducing cytokine production, we can conclude that immune dysregulation, especially those in innate immune system, is a primary, causal contributor to the development of neurodegenerative diseases.

Intriguingly, different neurodegenerative diseases are associated with distinct TREM2 variations. Homozygous mutations are associated with Nasu–Hakola disease (NHD),^189^ or frontotemporal dementia,^190^ while the TREM2 variants associates with AD are heterozygous.^191^ Different mutations in TREM2 seem to generate diverse affection on the function of it. Kober et al. found that mutations associated with NHD are buried inside the protein. These kinds of mutations affect the correct fold of TREM2 and the stability of it, leading to reduced presence of TREM2 on the membrane of immune cells. However, AD associated mutations are appeared on the surface of TREM2. These kinds of mutations were found to impair the binding ability of TREM2 to a group of its ligands, glycosaminoglycans.^192^ The AD associated TREM2 mutations seem to reshape the epitopes on the surface of TREM2, and reduce its binding with some ligands, thus remodel the response of TREM2 expression immune cells to the stimulus in the microenvironment. These results also indirectly demonstrate that TREM2 should be a receptor binding with multiple ligands and has complicated function. Numerous TREM2 mutations linked with AD have been identified, including R47H, R62H, N68K, D87N, T96K, and so on.^191,193^ R47H mutation confers the strongest risk to AD and is the most frequently studied variant in TREM2. Some studies have revealed that R47H variation reduced the presence of TREM2 on the surface of immune cells by preventing the maturation^194^ or by reducing the stability of TREM2.^195,196^ However, other studies found that R47H variant affected TREM2 function by altering its binding ability rather than expression.^192,197,198^ R47H mutation also leads to multiple changed in AD pathology. Mice carrying R47H variation displayed impaired binding of TREM2 with anionic and zwitterionic lipids which are known to associate with fibrillar Aβ. As a result, microglia failed to cluster around Aβ plaques and became apoptotic, subsequently leading to augmented Aβ accumulation,^198,199^ and spread of neuritic plaque tau aggregates.^200^ Indeed, increased phosphorylated tau and axonal dystrophy have been found around the amyloid plaques in AD patients carrying R47H variant.^201^ In line with these results, many studies have suggested that both the total tau and phosphorylated tau are increased in the CSF of R47H carrying AD patients compared with non-carriers,^202^ and the increasing of tau in CSF can be attributed to the pathology burden of tau in the brain.^203^ In addition, TREM2 deficiency was also suggested to affect the migration of microglia. A study using human microglia derived from induced pluripotent stem cells (iPSCs) revealed that TREM2 knockout reduced survival, phagocytosis and migration of microglia.^204^ However, since tau pathology happens much later than Aβ aggregation during the time course of AD, Gratuze et al. raised an objection to theory that loss of function of microglia carrying R47H variant is an inducement of tau pathology. Their results suggested that R47H variant reduced phosphorylated tau and attenuated neurodegeneration in the PS19 mouse model of tauopathy.^205^ Recently, some advances on the molecular mechanism of TREM2 R47H mutation in affecting the development of AD have been reported,^206–209^ although the exact mechanism is still elusive.

**Fig. 5:**
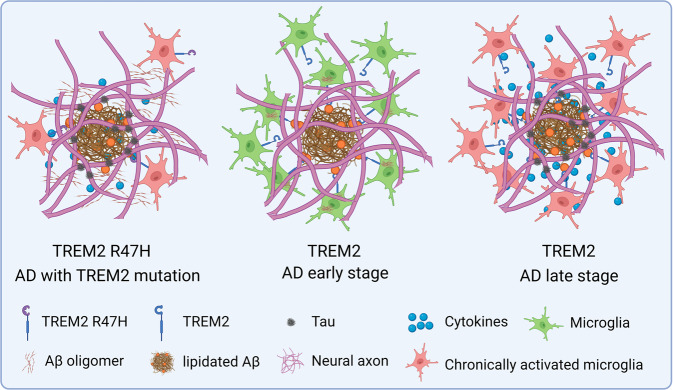
Function of TREM2 in AD. TREM2 expression enhances the proliferation and migration of microglia, and improves the phagocytosis ability. At the early stage of AD development, TREM2 expression microglia surround the Aβ plaque, interact with lipidated Aβ to efficiently clear them. The surrounding microglia also prevent the spread of Aβ. But at the late stage of AD, TREM2 expression microglia fail to clear the aggregates, leading to chronic inflammation, which then attenuates the phagocytosis ability of microglia, induces tau phosphorylation and aggregation. For individuals with TREM2 mutation, the affinity of TREM2 was weakened, which inhibits the proliferation and migration of microglia, leading to the failure of microglia surrounding Aβ aggregates. TREM2 mutation also enhance the secretion of cytokines from microglia. As a result, TREM2 mutation in microglia benefits the spread of Aβ, and the phosphorylation and aggregation of tau, exacerbating AD pathology

In the CNS, TREM2 can interact with a range of ligands, but it is mostly known as a receptor for lipid substrates.^210^ For example, APOE, ApoJ/CLU, Galectin-3, lipidated Aβ as well as lipids exposed on the surface of apoptotic cells can bind with TREM2 and activate it.^211^ In addition, in vitro study found that nucleotides released from damaged cells may also bind with TREM2.^212^ For AD patients without TREM2 variations, TREM2 signaling still plays crucial role in the progression of disease. Single-nucleus RNA sequencing (snRNA-seq) data on postmortem samples from AD patients and on samples from AD mice model also revealed that the expression of TREM2 was increased in AD pathology.^164,213,214^ Further studies suggested that TREM2 was prominently upregulated in microglia around the lesion regions in a disease progression-related manner,^215–217^ although the other studies revealed that TREM2 positive cells were recruited monocytes rather than microglia.^218,219^ Expression of TREM2 can be induced by several ways. Binding with ligands, such as Aβ, anionic lipids, and APOE4, can increase the expression of TREM2, and epigenetic modification also regulates TREM2 expression. Moreover, pro- or anti-inflammatory signals were shown to affect TREM2 expression.^220^ Ordinarily, TREM2 activation is considered as a beneficial response in that it supports microglia’s migration toward amyloid plaques, preventing amyloid spread^221–224^; so we raise the question of why the increased expression of TREM2 in microglia fails to inhibit disease progression. In APP/PS1 mouse model, lentivirus mediated TREM2 overexpression protected the mice against neuroinflammation, Aβ aggregation, and neurodegeneration only at young age,^225^ while no protection was found in mice with old age.^226^ In another study using transgenic human TREM2 overexpression 5×FAD mouse model, increasing TREM2 levels reduced the expression of many disease-associated microglial genes and upregulated lots of microglial genes related to phagocytosis and anti-inflammation. While the transgenic human TREM2 leads to the high expression of TREM2 at the early stage of mice development, the expression of endogenous TREM2 was only found to be elevated at the later stage of disease.^227^ This may explain why the overexpressed TREM2 found in AD patients didn’t prevent disease progression. However, studies using TREM2 deficient model got conflicting results. Using an APP/PS1 AD mouse model, Jay et al. found that TREM2 deficiency reduced Aβ deposition in the young APP/PS1 mice, but exacerbated amyloidopathy in old APP/PS1 mice.^228^ Using the same model, TREM2 deficiency was also found to eliminate macrophages, attenuating inflammation and amyloid/tau pathologies.^229^ In distinct tau mouse models, TREM2 deletion have yielded more diverse results.^230–233^ It seems that the role of TREM2 in AD is dosage- and time-dependent. Nonetheless, we still can glean some conclusions from these studies: the activation of microglia through TREM2 leads to their clustering around Aβ plaques, forming a neuroprotective microglial barrier that promotes amyloid compaction and insulation, restraining the accumulation of tau induced by Aβ plaques in early stages. However, the contribution of TREM2 during later stages of tauopathy is still in dispute, it seems that most of the results support an adverse effect of TREM2 at the later stage. Very recently, Jain et al. reported an interesting finding that chronic activation of TREM2 receptor with a TREM2 antibody increased the activation of peri-plaque microglia, and surprisingly aggravated tau pathology and neurodegeneration in a mouse model of amyloidosis in which tau was injected directly into the brain to induce Aβ-dependent tau seeding/spreading.^234^ It is possible that the adverse effect of TREM2 at the later stage of AD is caused by the chronic activation of it (Fig. 5). But further studies are needed to figure out why TREM2 deficiency mice yield conflicting results.

Recently, soluble TREM2 (sTREM2), generated by the proteolytic cleavage of full length TREM2 by ADAM10 and ADAM17, was found to change dynamically in the CSF of AD patients during the progression of disease, peaking at the early symptomatic stages of the disease.^211^ Epidemiologic study of AD patients revealed that increased sTREM2 was shown to be beneficial: it can inhibit the fibrillization of Aβ peptides, increase the uptake of Aβ fibrils into microglial cells,^235^ and attenuate cognitive and clinical decline in AD patients and AD mouse models.^236,237^ However, some studies also found that sTREM2 promoted microglial survival in a PI3K/Akt-dependent manner and stimulated the production of inflammatory cytokines depending on NF-κB in vitro.^238^

Although the exact molecular mechanism of TREM2 pathway is still far from being fully elucidate, some progress has been made in recent years. Mounting evidence demonstrated that DNAX activating protein of 12 kDa (DAP12) is an important candidate in this process. Study using in vitro cultured microglia suggested that binding of the ligand to TREM2 induces phosphorylation of the tyrosine residues within the immunoreceptor tyrosine-based activation motif (ITAM) of DAP12, recruiting spleen tyrosine kinase (Syk) to activate downstream signaling molecules, such as phosphatidylinositol 3-kinase (PI3K), phospholipase Cγ (PLCγ), extracellular signal-regulated protein kinase (ERK) among others.^239^ Although DAP12 is generally considered to induce activation signal through its ITAM domain, the TREM2/DAP12 complex can also induce inhibitory signals,^240,241^ JNK and ERK1/2 signaling have been suggested to be involved in this process.^240,242^ We still know little about how DAP12 regulates ERK1/2 and JNK signaling. More studies are needed to figure out the downstream molecular pathways of DAP12 activation. It was proposed that the dual functions of TREM2 signaling may be caused by DAP12 responding differently to ligands with different affinities. Ligands with low affinity induce anti-inflammatory signaling, while those with high affinity induce proinflammatory signaling.^243^ Recently, TREM2 was suggested to induce the expression of IL-3R receptor in microglia, which made microglia responsive to IL-3 secreted from astrocytes. Activation of microglia by IL-3 signaling improved the migration of microglia to the Aβ aggregates and enhanced the phagocytic ability of microglia.^244^ TREM2 mutations were also reported to be risk factor for PD and ALS.^245,246^

Except APOE and TREM2, large-scale genome-wide association study (GWAS) has identified many additional genetic risk factors linked with immune response (odds ratio 0.9–1.15). Interestingly, many of these immune molecules either express specifically in microglia (e.g., the MS4As, CD33, SPI1, and INPP5D) or are enriched in microglial cells (e.g., CR1, ABCA7, and CLU), suggesting that microglia plays crucial role in the immune regulation of AD.^247^ Some of the molecules have been reviewed recently.^248^ However, for most of the molecules identified by GWAS, further studies are needed to verify the role of these molecules in the development and progression of AD.

#### Parkinson’s disease

Multiple common genetic variants between PD patients and other inflammatory diseases have been identified.^249,250^ In addition, GWAS have identified several immune-related gene variants in PD,^251^ including leucine rich repeat kinase 2 (LRRK2), glucosylceramidase (GBA), parkin RBR E3 ubiquitin protein ligase (PRKN) and PTEN Induced Kinase 1 (PINK1).

LRRK2 mutation is the most prevalent cause of familial PD and happens in ~1% of sporadic PD.^252^ LRRK2 is expressed at low level in resting neuronal cells, including neurons, microglia, and astrocytes, but its expression is upregulated in neuronal cells and many immune cells (i.e., monocytes, macrophage, T cells, B cells) after stimulation by pro-inflammatory mediators, such as TNFα, IFNβ, IL-6, IFN-γ and LPS.^253^ LRRK2 has been found to promote neuron death and enhance immune response.^254^ So far, eight LRRK2 genetic variants have been identified in PD patients. G2019S mutation, the most common LRRK2 variant,^255^ has been suggested to increase the activity of LRRK2,^256^ leading to neuronal toxicity^257^ and increasing the level of proinflammatory cytokines in peripheral.^258^ In addition, for sporadic PD patients without LRRK2 mutations, the expression of LRRK2 was found to be increased in immune cells.^259^ Infection of G2019S carrying mouse with reovirus leads to elevated ROS production and α-synuclein concentration in the brain compared with non-carriers, probably through modulating inflammation.^260^ These results indicate that LRRK2 hyperactivation is an important immune-related genetic factor in PD.

Glucocerebrosidase, a lysosomal enzyme encoded by GBA gene, is crucial for the degradation of glucosylceramide. GBA mutation is the other genetic risk factor for PD. In addition, idiopathic PD patients usually display reduced GBA activity in their monocytes.^261^ In vitro study using an iPSCs derived macrophage revealed that GBA deficiency increased proinflammatory cytokine expression.^262,263^ In line with this, PD patients with GBA mutation have increased plasma levels of chemokines, including CCL2, CXCL8, and CCL3.^264^ Mice carrying GBA L444P mutation exhibited partial enzyme deficiency, which then led to multisystem inflammation.^265^ These results indicate that GBA dysfunction leads to immune dysfunction which may be responsible for the development of both idiopathic and GBA-associated PD.

Mutations in PRKN (which is encoded by PARK2 gene) and PINK1 (which is encoded by PARK6 gene) have also been identified in familial and sporadic PD.^266^ Both PRKN and PINK1 are involved in mitophagy. Multiple studies have revealed that loss of PRKN and PINK1 dysregulated mitophagy and increased mitochondria stress, leading to mito-inflammation,^267^ which was recently suggested to be involved in PD pathology in PINK1 and PRKN deficient mice.^268^ In addition to mito-inflammation, both PRKN and PINK1 have been implicated in adaptive immunity through repressing mitochondrial antigens presentation. In support of this, in vitro study revealed that PRKN or PINK1 knock out enhanced the presentation of mitochondrial antigen by dendritic cells. Intestinal infection of bacteria also led to enhanced mitochondrial antigen presentation and autoimmune response in PINK knockout mice compared with control,^269^ which may explain the increased CD8+ T cells in PD patients.^270^ In conclusion, loss of activities in PRKN and PINK1 during PD may increase mito-inflammation and enhance mitochondrial antigen presentation, promoting the development of PD. Besides the genetic mutations mentioned above, mutations in numerous other immune-related genes have been identified by GWAS, including VPS35, PARK7, HLA, GPNMB, TMEM175, PGRN. The roles of these genes in PD have been reviewed by some excellent reviews recently.^49,253,271^

#### Amyotrophic lateral sclerosis

In the ALS database, around 150 genes have been reported to have contribution to ALS pathogenesis. More than 30 of these genes strongly correlate with the disease, although their exact roles in disease development are still not completely understood.^85^ Many of the genetic mutations are associated with immune response, such as C9orf72, TBK1, CYLD, OPTN, PGRN, and so on.^85,272^ Most of them were suggested to induce mitochondrial impairment, which further led to oxidative stress and inflammation.^273^ These genes have been discussed in multiple reviews recently.^273,274^ We will not discuss them in depth.

## Signaling pathways that can induce inflammation in the CNS

In the CNS, multiple cells, including microglia, astrocyte, oligodendrocyte, endothelial cells, pericytes and so on, are involved in sustaining the homeostasis of the CNS. Microglia and astrocytes have multiple functions and play central role in the innate immune responses in the CNS. Traditionally, they were simply classified into two opposing phenotypes: M1/M2 (for microglia) or A1/A2 (for astrocyte). However, the recent scientific approaches, such as single-cell RNA sequencing, have revealed that these glial cells have multiple reactive phenotypes related to their regional location and the type and stage of neurodegenerative diseases they are in ref. ^275^ Microglia and astrocyte sense stimulus through cellular receptors on the surface (e.g., TLR, RAGE, cGAS), and secrete proinflammatory cytokines, chemokines, lipid mediators, NO and so on, to recruit additional immune cells and remove pathological agents. Generally, inflammation is a neuroprotective mechanism, but sustained chronic inflammation in the CNS causes neurotoxicity and promotes neurodegeneration. The sensors and inflammatory mediators can be used as the target to intervene glial cells activation. Understanding the molecular mechanism underlying these signaling pathways is important for proper design of the strategy for anti-inflammatory therapy of neurodegenerative diseases.

### TLR signaling

**Fig. 6:**
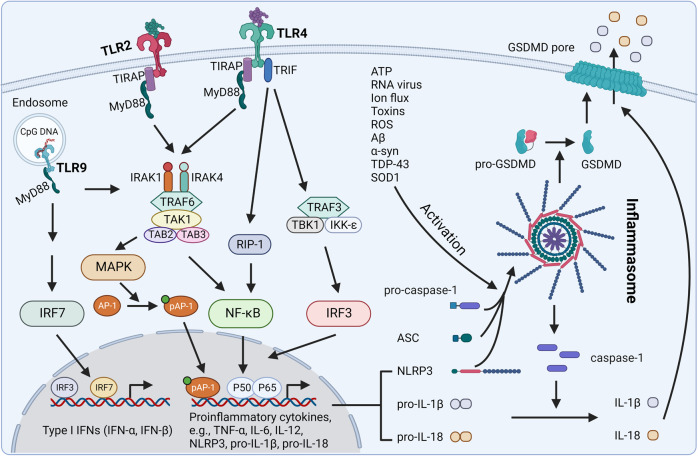
Crosstalk between TLRs and the inflammasome pathway. TLR2, TLR4, and TLR9 are the most involved TLR receptors in neurodegenerative diseases. The binding of aggregates (e.g., Aβ, α-synuclein) or other PAMPs or DAMPs activates TLRs. Activation of TLR2 induces the MyD88-dependent pathway, which activates MAPK and NF-κB, leading to the release of proinflammatory cytokines. In addition to the MyD88-dependent pathway, TLR4 activation also transduces signal through an MyD88-independent pathway, which leads to the activation of the IRF3 transcription factor and the subsequent release of type I IFNs. TLR9 is located on internal vesicles and binds to bacterial and viral nucleic acids or endogenous CpG DNA. Activation of TLR9 induces MyD88-dependent signaling, translocation of IRF7 into the nucleus, and the release of type I IFNs. TLR activation also induces the expression of NLRP3, pro-IL-1β, and pro-IL-18, acting as the first signal (or priming signal) for the NLRP3 inflammasome pathway. A variety of stimuli, including ATP, RNA virus, and aggregates, act as the second signal (or activation signal) activating NLRP3 and inducing the assembly of NLRP3, ASC, and pro-caspase-1 into an inflammasome. This process activates caspase-1, which in turn cleaves pro-IL-1β and pro-IL-18 into IL-1β and IL-18, respectively. NLRP3 inflammasome activation also induces the maturation of GSDMD, which translocates to the plasma membrane and forms a pore through which the cleaved IL-1β and IL-18 molecules can be released into the extracellular space. In addition, GSDMD can induce an inflammatory form of cell death termed pyroptosis. The cleaved cytokines have both autocrine and paracrine effects that further amplify the inflammatory response

TLRs, the best-characterized family of PRRs, are a group of receptors widely distributed across organisms and play crucial roles in innate immune responses. A total of ten TLRs in human and thirteen TLRs in mice have been identified. Several of them, especially TLR2, 4, and 9, have vital contributions to the development of neurodegenerative diseases.^276^ TLRs can be activated by pathogen-associated molecular patterns (PAMPs) (e.g., bacteria, viruses, or fungi) and DAMPs (e.g., protein aggregates, ATP, mtDNA).^277^ TLR activation leads to receptor dimerization and the recruitment of adapter proteins, such as Toll/interleukin 1 receptor (TIR) domain-containing adapter interferon-β (TRIF) and myeloid differentiation primary-response protein 88 (MyD88)^276^ (Fig. 6). TLR3 transduces signaling via TRIF, while TLR4 can signal through both TRIF and MyD88, and all other TLRs mediate through MyD88 adapters.^278^ For TLRs located on the cell surface (i.e., TLR1, 2, 4, 5, 6), once activated, MyD88 is recruited to the cell surface. In the case of TLR2 and TLR4, MyD88 recruitment is indirect and requires the help of toll-interleukin-1 receptor (TIR) domain-containing adapter protein (TIRAP). MyD88 forms a complex with IL-1R-associated kinases (IRAK) family proteins to recruit and activate tumor necrosis factor receptor-associated factor 6 (TRAF6). Activated TRAF6 induces the activation of TAK1 and TAB2/3, followed by the consequent activation of mitogen-activated protein kinase (MAPK) and nuclear factor kappa B (NF-κB), leading to a cascade of inflammatory responses.^279^ For intracellular TLRs (i.e., TLR7, 8, 9), their stimulation leads to the recruitment of MyD88, IRAK4, IRAK1, and TRAF6 in sequence, as well as the translocation of interferon-regulatory factor 7 (IRF7). TLR3 (intracellular receptor) and part of TLR4 signal through a MyD88-independent pathway. Once activated, their signals mediate through TRIF, which finally results in the translocation of NF-κB and IRF3 to the nucleus and induces the production of interferons (e.g., IFN-β) and chemokines (e.g., CCL5, CXCL10).^280^ TLRs are widely expressed in neural cells, including microglia, astrocytes, oligodendrocytes, neurons and so on.^281^ It has been reported that TLRs are upregulated in the brains of patients with neurodegenerative diseases.^282^

TLR2 is one of the most studied TLRs related to neurodegenerative diseases. In APP23 transgenic mice, TLR2 on microglia was shown to co-localize with Aβ plaques,^283^ and Aβ aggregates can bind to and activate microglia through the TLR2 receptor.^284^ Inhibiting of TLR2 signaling decreased activation glial cells, reduced Aβ deposition and prevented memory decline.^285,286^ In vitro and in vivo studies revealed that knock out TLR2 and TLR4 dissipated Aβ1-42 induced activation of macrophages and dendritic cells, and prevented memory decline induced by Aβ1-42 immunization.^287^ However, some studies yielded conflicting results. For example, it has been suggested that activation of TLR2 receptor enhances the uptake of pathological Aβ by in vitro cultured microglial cells.^288^ In an APP/PS1 transgenic AD mice model, TLR2 knockout aggravated white matter damage and neurobehavioral functions, this process may be mediated by excess astrocyte activation.^289^ These conflicting results may further demonstrate that TLR signaling can be beneficial or detrimental to the host. It is possible that TLR2 activation is beneficial for the clearance of Aβ aggregates, genetic deletion of TLR2 may aggravate the aggregation of Aβ, leading to persistent inflammation. At the same time, persistent TLR2 activation by Aβ, environment or genetic factors cause chronic inflammation, which is detrimental to neurodegeneration. Pourbadie et al. reported that intracerebroventricular injection of low-dose TLR2 ligands attenuated spatial and working memory disturbances, and restored long-term potentiation which was impaired by Aβ plaque.^290^ It is possible that a weak TLR2 signal has a beneficial role in neurodegenerative diseases. Taking together, TLR2 targeted therapy should inhibit its detrimental role while reserve its beneficial role.

It has been suggested that TLR2 expression is upregulated in PD postmortem human brains.^291,292^ α-synuclein released from neurons can act as an endogenous DAMPs, bind to TLR2, and then be endocytosed and transported to lysosomes for degradation or spreading to adjacent cells.^293^ α-synuclein can also activate glial cells by binding to TLR2^61,294,295^ and enhance the spreading of α-synuclein.^70,296^ TLR2 activation further enhances the aggregation of α-synuclein by regulating autophagy.^292^ In line with these results, blocking the interaction between TLR2 and MyD88 can reduce the activation of glial cells, decrease α-synuclein spreading, and protect dopaminergic neurons.^297^

TLR2 expression in microglia was also enhanced in ALS mouse model, and the increased expression of TLR2 was strongly associated with aggravated neuroinflammation and degeneration of motor neurons.^298^ TLR2 expression was also enhanced in the post-mortem spinal cord tissue from sporadic ALS patients.^299^ Overexpression of mutant SOD1 enhanced the activation of microglia, which was mediated by TLR2.^300^

TLR4 is also believed to be closely associated with neuroinflammation in neurodegenerative diseases. A single intracerebroventricular injection of Aβ into C57BL/6 mice induced activation of glial cells and impaired recognition memory, while an antagonist of TLR4 eliminated the neurotoxic effect of Aβ on memory. Moreover, Aβ had no effect on memory or glial cell activation in TLR4 knockout mice.^301^ Just several months ago, Meng et al. reported that aggregates formed by hyperphosphorylated tau can induce human macrophage activation in a TLR4-dependent manner.^46^ But some studies show conflicting results. In a tau pathology mouse model, chronic mild stimulation of TLR4 signaling with LPS was found to reduce tau aggregations and improve memory impairment.^302^ These conflicting results may reflect the complicated functions of TLR4 in neurodegeneration, which may be disease stage-dependent or signal strength-dependent.

TLR4 imbalance may also play an important role in α-synucleinopathies. TLR4 has been found to play important role in α-synuclein-induced microglia activation in vitro.^303^ The absence of TLR4 can reduce neuroinflammation through multiple pathways in an MPTP-induced PD mouse model.^304^ TLR4 antagonists significantly reduce the death of primary neurons co-cultured with glial cells, indicating the involvement of TLR4-mediated glial cell activation in α-synuclein oligomer-induced neuronal death.^305^ However, Venezia et al.’s study yields a conflicting result. They found that activation of TLR4 with an agonist in α-synuclein overexpressing mice increased uptake of α-synuclein by microglia, prevented neuronal degeneration and ameliorated motor deficits.^306^ In addition, gut microbial dysbiosis was suggested to alter TLR2 and TLR4 signaling, promoting α-synuclein aggregation in enteric and vagal neurons, which in turn migrates to the brain via peripheral nerves and contributes to neurodegeneration.^307^ These results provide a strong evidence for the involvement of gut microbial dysbiosis in PD development.

The impact of TLR4 on ALS has also been reported. An analysis of post-mortem tissue from sporadic ALS patients showed a increased TLR4 expression in the spinal cord,^299^ and immunostaining revealed that TLR4 is mainly expressed in astrocytes located in the white and gray matter of cervical spinal cord tissue from ALS patients.^308^ In the hSOD1 G93A transgenic mouse model, the expression of TLR4 was upregulated in microglia and astrocytes, and TLR4 deficiency or antagonist treatment decreased microglia activation, improved motor function and extended life expectancy.^309,310^

TLR9, the sole TLR that can detect DNA, was originally found as a sensor for bacterial DNA that has abundant unmethylated CpG dinucleotides.^311^ However, unmethylated, CpG-rich sequences are present in mammalian nuclear DNA, albeit at low frequency.^312^ There are also many unmethylated, CpG-rich sequences in mtDNA.^313,314^ Therefore, DNA released from damaged cells can also trigger sterile inflammation via TLR9,^315^ making TLR9 an important sensor in neurodegeneration. TLR9 was found to be overexpressed in the substantia nigra and putamen of PD patients and in striatum of a PD mouse model,^316^ as well as in the spinal cord of an ALS mouse model.^317^ DNA derived from degenerating neurons evokes NF-κB activation in microglia via TLR9.^318^ In vitro experiments revealed that CpG-DNA caused microglia activation through TLR9, which further induced neuronal toxicity.^319^ But CpG-DNA can induce neuronal degeneration in vivo only when glucocorticoid receptors (GRs) were removed, because decreased GRs, which is observed in the brain of PD patients, enhanced the activation of TLR9.^320^ Meanwhile, a neuroprotective role of TLR9 signaling in microglia has also been reported. Several studies have shown that TLR9 activation can enhance the clearance of aggregates and reduce AD-related pathologies in mouse and squirrel monkey models of AD.^321,322^

In conclusion, DAMPs (e.g., Aβ, tau, α-synuclein, CpG), environmental factors and genetic factors can activate glial cells through TLR receptors and alter their phenotypes, which could contribute to the development of neurodegenerative diseases. At the same time, TLR activation also promotes the clearance of DAMPs which may be beneficial to neurodegenerative diseases. TLRs should be potential targets for developing immuno-based therapies for neurodegenerative diseases. However, our current knowledge on their functions in glial cells and the underlying mechanisms is still limited. The diversity of TLRs and wide distribution of them make the research more difficult. In the future studies, specifically deletion of each TLR receptor in specific glial cells using animal models could help us to clarify the role of different TLRs in different pathological conditions. The diversity of TLRs and the overlap between the downstream signaling of TLRs increase difficult in TLRs targeted therapy.

### RAGE signaling pathway

RAGE belongs to the group of PRR receptors that can interact with DAMPs or PAMPs to induce an innate immune response.^323^ In the CNS, RAGE is expressed by multiple cell types, including microglia, astrocytes, neurons, pericytes and endothelial cells.^324^ Upon binding with the ligand, RAGE activates MAPK and PI3K signaling through its intracellular effector molecule, diaphanous 1 (DIAPH1). RAGE-DIAPH1 interaction increases the production of ROS and inflammatory cytokines, promotes cellular migration, and inhibits the expression of cholesterol transporters (e.g., ABCA1, ABCG1), thereby resulting in lipid accumulation and cellular dysfunction.^325^ In addition, RAGE was also suggested to induce MyD88-dependent proinflammatory signaling, with TIRAP acting as a bridge, which is similar to TLR signaling.^326^ RAGE is receptor for advanced glycoxidation end products (AGEs). Non-enzymatic glycation is the most common source of AGEs within the body.^327^ Diet is the second source of AGEs: high-heat processed grains, nuts, and canned meats are enriched in AGEs.^327,328^ An oxidized environment increases the production of AGEs,^329,330^ and RAGE activation further drives ROS production,^325^ which forms a feed-forward loop in which AGEs drive more AGEs. The activation of RAGE by AGEs generates systemic inflammation, which may promote the development of neurodegeneration. In aging and diabetes, the levels of RAGE ligands are increased in both the CNS and the periphery, which upregulate the expression of RAGE receptor and induces multiple downstream events.^324^ This may be a potential mechanism underlying the close association between aging/diabetes and neuroinflammation. Increased RAGE Ligand burden and excess RAGE activation also occurs in neurodegenerative diseases. Moreover, GWAS found mutations within RAGE which was suggested to increase its affinity to ligands and enhance inflammatory response after activated by its ligand.^325^ Aβ aggregates can act as DAMPs and induce RAGE-dependent inflammation signaling. Yan et al. reported that Aβ peptides and Aβ oligomers can bind to RAGE receptor and activate glial cells.^331^ Blocking the interaction of Aβ with RAGE inhibits microglial activation and reduces the release of proinflammatory cytokines.^332^ RAGE is also reported to play an important role in the uptake of Aβ by microglia.^333^ The binding of Aβ to RAGE mediates Aβ transport from the cellular surface to mitochondria in microglia. Aβ shuttling into mitochondria exacerbates mitochondrial dysfunction, which induces NLRP3 inflammasome activation.^334^ In addition to AGEs and Aβ, other molecules that can bind with RAGEs have been reported, including advanced oxidation protein products (AOPP), members of the S100/calgranulin protein family (e.g., S100B), HMGB1, and heat shock protein 70 (HSP70), among others.^335^ These molecules can be released from damaged neurons and act as DAMPs to trigger inflammation. The expression of HMGB1 and S100B was increased in neuron and glial cells in many neurodegenerative diseases, leading to excessive release of them and activation of RAGEs. In vitro and in vivo studies revealed that silencing of HMGB1 or S100B reduced neuroinflammation and attenuated neurodegenerative diseases.^336,337^ RAGEs receptor was also highly expressed in PD patients,^338^ and RAGE gene polymorphisms were associated with sporadic PD in the Chinese Han population.^339^ Deficiency of RAGE improved the survival of dopaminergic neurons in an MPTP-induced PD mouse model.^337,340^ The RAGE pathway is also involved in the pathogenesis of ALS. The levels of RAGE and its ligands (e.g., AGEs) were increased in the spinal cord and cerebrospinal fluid (CSF) of ALS patients.^299,341^ A transcriptomic analysis of cervical spinal cord from ALS patients revealed that RAGE expression was negatively correlated with disease severity and the age of disease onset. The RAGE expression level was closely linked to signaling pathways related to extracellular matrix composition, cell-cell communication and lipid metabolism.^342^ Whole-body knockout of RAGE reduced inflammation and extended the survival in an hSOD1 G93A transgenic mouse model.^343^ However, the microglia-specific knockout of RAGE at disease onset extended the survival of only male, and not female, hSOD1 G93A transgenic mice.^342^ Moreover, a conflicting result was also reported. Liu et al. found that RAGE haploinsufficiency in hSOD1 G93A transgenic mice can extend their survivals, but RAGE complete knockout showed no benefits.^344^

In summary, accumulation of RAGE ligands and excessive RAGE activation increase cellular stress, impair lipid and cholesterol handling, enhance ROS production, which again amplifies the accumulation of ligands, forming a feed-forward loop of inflammation in glial cells, endothelial cells, myeloid cells etc. At present, most of the mechanistic studies of RAGE signaling in neurodegeneration have been conducted in animals with global deletion of RAGE. Therefore, further studies using cell type- and temporal specific RAGE knockout animal models would be important to clarify the function of RAGE in neurodegenerative diseases. Multiple small molecules have been developed to block RAGE signaling and have shown potential to inhibit glial cells activation in vitro and in animal models.^345^ But they still need further pre-clinical and clinical studies to justify whether it could be useful in the treatment of neurological disorders.

### Inflammasomes

Inflammasomes are cytosolic multiprotein complexes consisting of the cytosolic sensor protein, the adapter apoptosis-associated speck-like protein containing a caspase recruitment domain (ASC), and pro-caspase-1.^346^ Upon activation, the PRR protein, i.e., the sensor protein, undergoes oligomerization, followed by the recruitment of pre-existing pro-caspase-1 with or without the help of adapter protein, leading to autoactivation to generate active caspase-1. Subsequently, caspase-1 will process the biologically inactive pro-IL-18 and pro-IL-1β into active cytokines. Moreover, caspase-1 also induces pyroptosis, which is characterized by gasdermin D (GSDMD) mediated plasma membrane rupture.^347^

Numerous inflammasomes have thus far been identified. NLRP1, NLRP2, AIM2, NLRC4 and NLRP3 have been suggested to be involved in the progression of neurodegenerative diseases. NLRP3 is the most studied inflammasome, with a broad spectrum of activating stimuli, including PAMPs (e.g., bacterial, viral, and fungal) and DAMPs (e.g., ATP, uric acid, and aggregates).^348,349^ Activating NLRP3 is a very complex process, which makes it unique among the inflammasomes. Moreover, our knowledge of the mechanisms responsible for NLRP3 activation is still limited. Currently, a two-signal model has been proposed for NLRP3 signaling. The first signal (or priming signal) is triggered by binding of cytokines or microbial components to TLRs or the corresponding receptors of specific cytokines, leading to activation of NF-κB which then increases the expression of pro-IL-1β and NLRP3. The second signal (or activation signal) is provided by a range of stimuli, including ATP, RNA viruses, pore-forming toxins, and so on. Many molecules or cellular events, including ROS, ionic flux, mitochondrial dysfunction and lysosomal damage, have also been shown to activate NLRP3^350^ (Fig. 6). The necessity of the first priming step to assemble the inflammasome and activate NLRP3 suggests that immune cells need a signal indicating either the presence of infection or the presence of proinflammatory cytokines to license themselves to sense danger signals in their immediate environment and activate the NLRP3 signaling pathway. That priming step thus prevents accidental or uncontrolled NLRP3 activation. Because the wide-ranging stimuli for NLRP3 (e.g., ATP, toxins, RNA virus, K^+^ ionophores) are structurally and chemically dissimilar,^351^ it is unlikely that NLRP3 binds to these stimuli directly. Instead, NLRP3 may sense a common cellular event induced by different stimuli.

In 2008, NLRP3 inflammasome was first found to be involved in AD. Halle et al. found that fibrillar Aβ can induce the secretion of IL-1β which depends on the presence of NLRP3.^352,353^ In APP/PS1 mice model, NLRP3 knockout reduced Aβ deposition by enhancing phagocytic clearance capacity and increasing extracellular Aβ degradation by insulin- degrading enzyme (IDE).^354^ Using the same model, Dempsey et al. found that deletion of NLRP3 or caspase-1 promoted microglia to anti-inflammatory phenotype, with reduced expression of IL-1β and caspase-1.^355^ These data suggest NLRP3 to be a potential target for AD therapy. Indeed, MCC950, a specific NLRP3 inhibitor, showed therapeutic effect in many neurodegenerative animal models.^355,356^ However, MCC950 is still not approved for the clinical usage as therapeutic method of neurodegenerative diseases. Formation of ASC specks is a typical feature of inflammasome activation. Pyroptosis of glial cells leads to release of ASC specks into the extracellular space, where it binds rapidly to Aβ peptides, increasing the aggregation of Aβ.^357^ In addition, the core of Aβ plaques in the brain tissue of AD patients can be stained by ASC. This is an interesting finding, indicating that pyroptosis of immune cell and subsequent release of ASC speck play important role in the earliest stage of Aβ deposition and AD progression. Thus, ASC speck should also be a potential target for AD therapy.

NLRP3 was also related to the pathogenesis of PD. In an in vitro experiment, both monomeric and fibrillary α-synuclein can stimulate TLR2 and induce the expression of pro-IL-1β, but only fibrillary α-synuclein can activate the NLRP3 inflammasome, resulting in the secretion of active IL-1β.^358^ Peripheral blood mononuclear cells from PD patients showed activation of NLRP3 inflammasomes and increased secretion of IL-1β in the plasma.^359^ NLRP3 knockdown or knockout was also found to reduce neuroinflammation and neurodegeneration in an MPTP-induced PD mouse model.^360,361^ In a different PD mouse model, blocking IL-1β signaling using an IL-1 receptor antagonist also significantly attenuated neurodegeneration.^362,363^ On the other side, inflammasome activation also enhances the aggregation of α-synuclein. It was suggested that inflammasome activation enhances the truncation of α-synuclein by caspase-1 enzyme, increasing the tendency of α-synuclein to aggregate.^364^ Thus, inflammasome activation might form a feed forward loop that leads to uncontrolled α-synuclein aggregation and persistent chronic inflammation, which further causes neurotoxicity. However, the possible role of NLRP3 in PD development still needs to be clarified, since some studies found no changes in IL-1β and IL-18 serum levels in PD patients compared with healthy controls.^347^

Several studies have also revealed the function of the NLRP3 signaling in the pathogenesis of ALS. Italiani et al. found that the concentration of IL-18 is increased in the serum of ALS patients, while no changes in the concentration of IL-1β can be detected.^365^ In line with this finding, ALS patients are also found to be associated with increased expression of NLRP3 and caspase-1 in the brain.^366^ Moreover, the expression of NLRP3 and ASC was greatly increased in the anterodorsal thalamic nucleus of ALS mouse model.^367^ The NLRP3 inflammasome in microglia was also involved in TDP-43-induced toxicity to motor neurons.^83^ Administration of 17β-estradiol, an anti-inflammatory molecule, decreased the expression of caspase-1 and NLRP3 and improved the survival of motor neurons in the lumbar spinal cord in ALS mouse model.^368^ In contrast, treatment of ALS animal model with IL-1R antagonist failed to attenuate neuroinflammation, indicating that NLRP3 may not play a critical role in ALS or the proinflammatory role of NLRP3 in ALS is mediated by IL-18, but not IL-1β.

At present, three inflammasome signaling targeted therapies, including anakinra, canakinumab and rilonacept, have been approved by US FDA for treating some inflammatory diseases. But none of them have been tested in clinical trials for the treatment of neurodegenerative diseases until now. The poor BBB penetration ability of these molecules may be the reason that restricts the usage of them in neurodegenerative diseases. NLRP3 targeted compounds, like MCC950, also exhibited therapeutic efficacy against several neurodegenerative diseases in preclinical trial. However, no compound was approved for the treatment of neurodegenerative diseases. Besides NLRP3, IL-1β can be induced by many other inflammasomes. Further studies need to compare the therapeutic effect between NLRP3 specific inhibition and the downstream inflammatory molecule blocking.

### Purinergic signaling pathway

The concept of purinergic signaling, coined by Geoffrey Burnstock in 1972, refers to cell signals that are activated by the binding of nucleosides or nucleotides to specific receptors.^369^ Purinergic receptors can be divided into two large families: P1 receptors for adenosine and P2 receptors for nucleotides, such as adenosine 5′-diphosphate (ADP) and ATP. P1 receptors include four members: A1, A2A, A2b, and A3, while P2 receptors can be subclassified into ionotropic P2X receptors and metabotropic P2Y receptors. The P2X family includes seven members (i.e., P2X1–P2X7), and the P2Y family contains eight members (i.e., P2Y1, P2Y2, P2Y4, P2Y6, and P2Y11–P2Y14). P2X receptors only respond to ATP, while P2Y receptors respond to ATP, ADP, uridine triphosphate (UTP), uridine diphosphate (UDP) and UDP-glucose.^370^ ATP is stored in millimolar concentrations in cells^371^ and may be released extracellularly through the damaged cell membrane or by various transporters. Th ectoenzymatic breakdown of released ATP produces most ADP, adenosine monophosphate (AMP), and adenosine in the extracellular space, although some studies revealed that adenosine can also be released directly by some neurons and astrocytes.^372^ Purinergic signaling is a dynamic, complex, multi-cellular process, involving ATP release, ectonucleotide enzyme-regulated generation of ATP derivatives (e.g., ADP, adenosine), and signal response of the purinergic receptors expressed on different CNS cells in a spatially and temporally heterogeneous manner. For example, excess ATP in neurons can be released at the synapse, where they are cleaved by CD39 (which is expressed on the surface of microglia) and CD73 (expressed on the surface of all the CNS cells) into adenosine, subsequently leading to adenosine/A1 receptor activation and synapse impairment.^373^

P2X7, which is widely expressed by immune cells, is the most studied purinergic receptors involved in neurodegeneration.^374,375^ Activation of P2X7 needs a high concentrations of ATP (mM level), which is much higher than the concentrations for other purinergic receptors (μM level).^376^ ATP’s activation of P2X7 induces Ca^2+^ influx and K^+^ efflux. The high intracellular concentration of Ca^2+^ induces the formation of nonselective plasma membrane pores by P2X7 and pannexin-1, leading to the drain of additional ATP into the extracellular space. K^+^ efflux can be sensed by NLRP3 and induces the assembly and activation of the NLRP3 inflammasome, leading to pyroptosis and more ATP release, activating other surrounding immune cells, and recruiting more immune cells from peripheral. Thus, activation of P2X7 by ATP forms a positive feedback loop, inducing consistent inflammation and leading to neurodegeneration.^377^ In addition, Ca^2+^ release also induces the production of ROS, thus changes the redox homeostasis of cells. Accumulation of ROS further leas to oxidation of proteins, DNA and lipids, thereby disrupting the function of mitochondria and triggering cell death.

It has been reported that Aβ1-42 can activate microglia through the P2X7 pathway, probably by binding directly to the P2X7 receptor.^378^ Mounting evidence has shown that there is an enrichment of P2X7-positive microglia around the senile plaques in human AD postmortem brain samples and AD mouse model.^379,380^ In vitro culturing of human microglia with amyloidogenic Aβ1-42 significantly improved the expression of P2X7. Aβ1-42 treatment also increased the intracellular Ca^2+^ concentration, membrane permeabilization, and release of ATP and IL-1β, all of which were dependent on the P2X7 receptor.^378^ Blocking or deleting the P2X7 receptor in multiple AD mouse models reduced neuroinflammation, diminished leakiness of the BBB, and attenuated memory impairment and cognitive deficiency.^381,382^

It has been shown that the expression and function of P2X7R are enhanced in PD patients.^383^ In a 6-OHDA rat model, P2X7 can be detected mainly in microglia but also in astrocytes. An antagonist for P2X7 significantly prevented the depletion of striatal dopaminergic neurons.^384^

The role of purinergic signaling in ALS has also been proposed. Brain samples from ALS patients have an increased expression of P2X7 in microglia.^385^ In hSOD1 transgenic mouse model, the expression of P2X7 is upregulated and the activities of ATP hydrolyzing are inhibited in microglia, further indicating the enhancement of P2X7 signaling during ALS development. P2X7 activation in astrocytes from the spinal cord induced a neurotoxic phenotype, which led to the death of motor neurons. Inhibiting P2X7 or increasing the degradation of extracellular ATP abolished the toxicity of astrocytes toward motor neurons.^386^ However, the disease progression in P2X7 knockout mice showed the opposite result: knockout of P2X7 increased astrogliosis, microgliosis, motor neuron loss, and proinflammatory cytokine secretion. These results show that constitutive deletion of P2X7 aggravates ALS pathogenesis, indicating that the P2X7 signaling may have a dual role in the development of ALS and the importance of time window for therapeutic intervention.^387^

Recently, P2X7 was found to be a scavenger receptor in the absence of ATP, while excessive ATP abolished P2X7 mediated phagocytosis.^377^ Given the critical role of phagocytosis in the pathogenesis of neurodegenerative diseases, these results suggest that P2X7 signaling might be beneficial to neurodegenerative diseases in the absence of excessive level of ATP. Under pathological condition, the excess ATP binds to P2X7 and activates NLRP3 inflammasome, inducing the pyroptosis of immune cells, which leads to release of more ATP. At the same time, phagocytic ability of immune cells is weakened. At this point, P2X7 activation forms a positive feedback loop, greatly promoting the progression of neurodegeneration.

### cGAS-STING pathway

Recognizing misplaced or foreign nucleic acids is one of the major ways the immune system protects human against pathogenic substances. In 2008, the cyclic GMP-AMP synthase (cGAS)-stimulator of interferon genes (STING) DNA-sensing pathway has been discovered as a vital mediator in detecting the misplaced or foreign DNA and triggering the release of type I interferons and other immune mediators.^388,389^ cGAS can sense cytosolic DNA,^390^ allosterically activate its catalytic activity and induce the synthesis of second messenger molecule, 2′3′ cyclic GMP–AMP (cGAMP).^388^ cGAMP produced by cGAS activation binds to STING which is located on the membrane of endoplasmic reticulum (ER)^391^ and leads to the translocation of STING from ER to the Golgi where it is further modified.^392^ On reaching the Golgi compartment, STING then recruits TBK1, which then activates IRF3 and NF-κB, leading to the expression of interferon-stimulated genes (ISGs), type I interferons and other inflammatory cytokines (e.g., IL-6, IL-12).^393–396^ cGAS-STING activation also causes lysosome cell death (LCD) and K^+^ efflux, which is one of the second signal of NLRP3 inflammasome as we discussed above, leading to the activation of NLRP3 and cell pyroptosis.^397^ At the same time, cGAS-STING signaling induces autophagy which is important for the clearance of intracellular DNA and virus.^398^ Considering the critical role of autophagy in neurodegeneration,^399^ further studies are needed to figure out the role of cGAS-STING mediated autophagy in neurodegenerative diseases. Besides the exogenous dsDNA released by virus or bacterial, intrinsic self-dsDNA released into the cytosol from nucleus or mitochondrion due to cellular or mitochondrial stress can also bind to cGAS and activate cGAS-STING pathway.^400,401^ Binding with long dsDNA has been shown to induce the rearrangement of cGAS to form a ladder-like networks that stabilizes cGAS-dsDNA complexes, which are considered critical for triggering inflammation.^402^ In contrast, activation of cGAS by short dsDNA is prevented. The ability of cGAS to distinguish shorter dsDNA from long dsDNA should be important for its specifically recognizing and responding to the “danger DNA”.

Mounting recent studies implicate the important role of cGAS-STING signaling in the development of neurodegenerative diseases. Neuroinflammation and senescence in AD was found to be mediated by cGAS-STING pathway. Nicotinamide riboside (NR), a NAD^+^ precursor, has been suggested to decrease the activation of glial cells partly through regulating the cGAS-STING signaling.^403,404^ Jin et al. reported an interesting finding that AD pathogenic protein tau can bind to polyglutamine binding protein 1 (PQBP1) and trigger inflammation in the CNS by activating cGAS-STING pathway.^405^ Recently, Xie et al. found that cGAS-STING pathway microglia was activated in aged mice and human AD. Knockout of cGAS in 5×FAD mouse largely protected from cognitive impairment and Aβ pathology,^406^ highlighting cGAS-STING as an important therapeutic target for AD. In contrast, STING stimulator, cGAMP, was found to induce triggering receptor expressed on myeloid cells 2 (TREM2) expression, which has been suggested to decrease Aβ deposition and improve cognitive impairment in AD.^407^ So the exact function of cGAS-STING signaling in AD is still far from understanding, studies in the future need to clarify the role of different face of cGAS-STING in affecting the development of AD.

cGAS-STING pathway is also related to PD pathogenesis. α-synuclein pathology was found to cause STING-dependent neuroinflammation and dopaminergic neurodegeneration.^408,409^ In PINK1 or PRKN deficient mice, mitochondrial stress induces mtDNA accumulation and increases the secretion of cytokines into the bloodstream which can be reversed by STING deletion. Knockout of STING reverses motor deficit and neuron loss in aged PRKN knockout PD mouse model.^268^ In addition, an important study published recently found that mice carrying STING N153S, a constitutively active STING, was sufficient to induce α-synuclein aggregation and degeneration of dopaminergic neurons.^410^ This study shows up the crucial role of cGAS-STING pathway in the development of PD. In this study, Szego et al. used a whole-body transgenic mice, which was impossible to identify the effect of constitutively active STING in specific cell type. In further studies, persistent activation of STING in specific cell type will be required to verify the role of cGAS-STING pathway in different cell types in neurodegeneration. Genetic variations in C9orf72 is the most prevalent cause of familial ALS and FTD. C9orf72 knockout myeloid cells are selectively hyperresponsive to activators of STING indicating that STING hyper-activation may be a cause of ALS and FTD.^411^ TDP-43 aggregates can also mislocate into mitochondria and cause mtDNA release, leading to STING dependent upregulation of inflammatory cytokines.^81^

Together, cGAS-STING activation occurs in response to a wide array of stimulus and generates multiple affection to the cell, including interferon response, NFκB and NLRP3 activation, LCD and pyroptosis mediated cell death, as well as enhanced autophagy. The multifaceted role of STING in specific cell type during the progression of neurodegenerative diseases remains to be determined which is important for the appropriate application of STING targeted therapy.

### Bioactive lipids

Activated immune cells secret multiple soluble mediators, such as cytokines and chemokines, that act as executors of inflammatory reactions. Numerous cytokines and chemokines have been shown to contribute to the pathogenesis of neurodegenerative diseases as summarized by the recent reviews.^9,253,412^

In addition to cytokines and chemokines, bioactive lipids also play crucial role during all phases of the innate immune responses. Bioactive lipids, which are generated from ω-6 or ω-3 essential polyunsaturated fatty acids (PUFA) precursors, can be divided into four main families according to their biochemical functions, including classical eicosanoids, specialized pro-resolving mediators (SPMs), phospholipids/sphingolipids and endocannabinoids (eCBs). Classical eicosanoids, derived from oxidation of ω-6 PUFAs, represent the most distinguished family of bioactive lipids. Eicosanoids allow the innate immune cells to respond rapidly to invaders, such as bacterial or amyloid plaques. Eicosanoids can be subclassified into multiple subfamilies, including the prostaglandins (PGs), thromboxanes (TXs), leukotrienes (LTs), hydroxyeicosatetraenoids (HETEs), lipoxins (LXs) and epoxyeicosatrienoids.^413,414^ Most of them act as initiators of inflammation. Upon activation of TLR4 by bacterial LPS and other stimuli, membrane phospholipids are cleaved by PLA2 to release free arachidonic acid (AA), a ω-6 PUFA, into the cytosol, which is then further metabolized to oxygenated derivatives eicosanoids by cyclooxygenase (COX), lipo-oxygenase (LOX) and CYP450 enzymes. COX is responsible for the synthesis of pro-inflammatory AA metabolites, including PGs and TXs, while oxidation of AA by LOX yields pro-inflammatory LTs, HETEs and anti-inflammatory LXs; oxidation of AA by CYP450 generates proinflammatory HETEs and anti-inflammatory epoxyeicosatrienoids.^414^ As we know, the prominent mechanism of NSAIDs, the widely used anti-inflammatory drugs, is to suppress the activity of COX.^415^ Oxidation of ω-3 PUFAs like docosahexaenoic acid (DHA) or eicosapentaenoic acid (EPA) by LOX or CYP450 leads to the generation of protectins, resolvins and maresins.^416^ LXs, protectins, resolvins and maresins are SPMs generated in human tissues and play critical role in inflammation resolution.^417^ Resolution is a highly coordinated process that occurs in response to inflammation to limit tissue damage and promote repair.^418^ It is widely accepted that resolution processes are initiated early during inflammation. Some pro-inflammatory lipids, such as PGE2, were shown to reduce the synthesis of leukotrienes and induce the production of SPMs by leading to lipid-mediator class switch at the peak of acute inflammation, thus the very same cells involved in the production of pro-inflammatory lipids start to produce SPMs.^419^ Some enzymes may also be involved in this process. COX2, one of the two known protein isoforms of cyclooxygenase, generally catalyzed the synthesis of pro-inflammatory lipid mediators, but acetylated COX2 changed its function to induce the synthesis of SPMs in microglia and resolve inflammation in AD mouse model.^420^ Failure of resolution is an important underlying cause of multiple chronic inflammatory diseases.^26^ The levels of many enzymes involved in the generation of prostaglandins or thromboxanes and the receptors for prostaglandins or thromboxanes have been found to be upregulated in neurodegenerative diseases.^421,422^ The affection of prostaglandins and thromboxanes signaling on neurodegeneration has been reviewed recently.^423,424^ Here we will mainly focus on the recent development on SPMs.

Numerous studies have identified abnormality of inflammation resolution in AD, including the abnormality in the profile of SPMs and its receptors.^175,425,426^ Multiple SPMs involved in AD have been identified, including lipoxin A4 (LXA4), maresin 1 (MaR1), protectin D1 (PD1), resolvin D1 (RvD1) and resolvin E1 (RvE1).^427^ Meanwhile, some of their receptors have been identified. Liquid chromatography-tandem mass spectrometry (LC-MS-MS) analysis of lipid mediators in the entorhinal cortex of AD patients revealed the downregulation of SPMs including protectin D1 (PD1), maresin 1 (MaR1) and resolvin (RvD5), and upregulation of PGD2, a proinflammatory lipid mediator, compared with age-matched controls.^428^ Analysis of postmortem hippocampal tissue from AD patients also found the association between reduction of SPMs and cognitive decline.^425^ Intraperitoneal injection of LXA4 and RvE1 alone or in combination into 5×FAD mice model increased the concentration of LXA4, RvE1 and RvD2 in the hippocampus, reduced the activation of glial cells, decreased Aβ accumulation.^429^ In vitro and in vivo studies suggested that MaR1 can inhibit inflammation and promote phagocytosis in microglia^428,430,431^ and improve the cognitive decline in AD mouse model.^432^ PD1 was also found to decrease inflammatory signals, suppress the production of APP by activating α-secretase and suppressing β-secretase in vitro.^433,434^ Combined administration of the SPMs, including MaR1, RvD1, RvD2, RvE1 and PD1, dramatically decreased microglial activation, ameliorated memory deficits and gamma oscillation impairments in the AD mouse model.^435^ In summary, many SPMs have been shown to affect AD progression through reducing activation of glial cells and enhancing phagocytosis of Aβ. But most of the studies are in vitro, and we still have limited knowledge on the underlying molecular mechanism. Further studies are needed to verify the role and mechanism of the SPMs in AD mouse model and patients.

SPMs were also suggested to be involved in PD and ALS, but their roles in PD and ALS remain largely unexplored. RvD1 was found to be decreased in human α-synuclein overexpression PD rat model and PD patients at early stage, early RvD1 administration inhibited inflammation in the CNS and peripheral, attenuated neuronal dysfunction.^59^ In a LPS induced rat PD model, injection of RvD2 into substantia nigra pars compacta suppressed the production of proinflammatory cytokines and reversed neural injury.^436^ In addition, Annexin A1 (AnxA1), one of the SPMs, was suggested to be a genetic risk factor for early onset PD, although such mutations are very rare.^437^ So far, there are still limited reports on the role of SPMs in PD. In an in vitro study, treatment of macrophage or mononuclear cells isolated from ALS patients with RvD1 significantly reduced the secretion of proinflammatory cytokines and chemokines.^438^ In a different in vitro study, MaR1 was found to reduce ROS production and NFκB activation, thus protect neuron from death.^439^

It must be noted that pro-resolution is different from anti-inflammation. Anti-inflammation reagents inhibit the factors that drive inflammation, including prostaglandins, cytokines, chemokines and so on. NSAIDs are representative of this kind of drugs. This kind of intervention dampens inflammation from the onset, which not only inhibits pro-inflammation process, but also sometimes also suppresses resolution. By comparison, pro-resolution reagents enhancing the factors essential for removal of the inciting stimulus as well as dampening pro-inflammatory signaling, followed by clearance of the apoptotic immune cells.^440^ In conclusion, attenuating inflammation itself might also interfere with successful resolution, since the process of resolution is initiated already very early during the inflammatory process.^441^ In other words, anti-inflammation therapy may reduce inflammation, but probably also blocked the full resolution of inflammation, suppressing the clearance of toxicity and inhibiting the recovery. In addition, many enzymes (e.g., COX and LOX) involved in the generation of pro-inflammatory lipids are also important for the biosynthesis of SPMs.^442^ These kinds of anti-inflammatory drugs may interfere with beneficial effect of SPMs too. Again, anti-inflammatory therapies might diminish the host response against pathogenic challenges.^440^ Thus, it should be better to intervene the progression of inflammation related disease by using SPMs than anti-inflammatory reagents. To develop anti-inflammatory drugs, it also should be careful to select targets to avoid the interference on pro-resolution pathways. These aspects have been reviewed in detail recently.^443,444^

### NO

NO is a signal molecule that regulates multiple physiological functions, mainly in the nervous system and cardiovascular system.^445^ It is generated during the conversion of L-arginine to L-citrulline with the catalysis of NOS. NOS is widely expressed in immune cells and non-immune cells.^446^ At present, three types of NOS have been identified, including neuronal NOS (nNOS, also named as NOS1), endothelial NOS (eNOS, also named as NOS3), and inducible NOS (iNOS, also named as NOS2).^447^ nNOS and eNOS, which are expressed constitutively, produce a low level of NO for short periods (seconds to minutes).^447^ iNOS is generally expressed by activated immune cells, leading to the generation of excessive levels of NO for long periods (hours).^445^ It is generally considered that low level of NO is crucial to sustain homeostatic functions, while excess level of NO is detrimental.^448^ So, to attenuate NO induced toxicity, iNOS is usually specifically targeted to avoid the disruption on the physiological functions of NO. But it was also suggested that excess NO during inflammation may induce the apoptosis of proinflammatory immune cells which was then cleared by immune-suppressive macrophage, indicating high NO level as a mediator of pro-resolving process.^449^ This makes targeting NO biosynthesis as a new therapeutic strategy a daunting task. For example, tumor associated macrophages or neutrophils were found to release NO which contributes to the apoptosis of CD8+ T cells in the tumor microenvironment.^450,451^ Systemic administration of NO inhibitors (iNOS inhibitors or eNOS inhibitors) attenuated pleurisy, but intrapleural administration aggravated inflammation probably through preventing inflammatory resolution.^452^

iNOS is expressed in both microglia and astrocytes.^453^ Excessive NO production in the CNS is a common pathological feature of neurodegenerative diseases.^445^ In AD, activation of microglia or astrocytes induces the expression of iNOS and leads to the release of excessive NO and ONOO^−^, which then promote the nitrotyrosination of Aβ_42_, facilitating the formation of Aβ plaques.^454,455^ What’s more, excessive NO and ONOO− lead to oxidative damage to biomacromolecules (proteins, lipids and DNA), resulting in the deposition of cytotoxic substances and the damage of mitochondria, ultimately enhance inflammation and induce neural apoptosis.^456,457^ This forms a positive feedback loop, benefiting the formation of chronic inflammation. iNOS deficiency strongly reduced the nitrotyrosination of Aβ_42_, attenuated Aβ aggregation and cognitive deficit in AD mouse model.^454^ Using a similar model, Nathan et al. found that the deficiency of iNOS also reduced activation of glial cells, protected the AD-like mice against premature mortality.^458^ But some studies got a conflicting result. In a APPSwDI transgenic mouse model, iNOS deficiency results in significant neuron loss in disease-relevant regions and further cognitive decline in behavioral tests despite unaltered levels of Aβ. iNOS deficiency also caused significant tau pathology.^459^ Difference of the transgenes used to build AD mouse model may be an explanation to this conflicting results. But it also demonstrates the complexity of NO signaling. Therapy strategies targeting NO should always keep the double-faced character of NO signaling in mind.

NO signaling is also involved in PD. Analysis of post-mortem human brain samples revealed increased levels of iNOS PD.^460^ In addition, iNOS expression was also found to be enhanced in multiple PD animal models induced by 6-OHDA,^461^ MPTP,^462^ and aSyn oligomers.^463^ NOS inhibition protected neuron from death in PD mouse models.^464–467^ These findings indicate that NO signaling is enhanced in PD and intervention of NO signaling may have potential for the treatment of PD.

### Immune signals from other cells

As we have discussed above, glial cells are the primary cells involved in neuroinflammation and neurodegeneration. Their crucial function in the development and progression of neurodegenerative diseases has also been elaborated in many reviews in the past year.^275,468,469^ Besides microglia and astrocytes, other cells, including endothelial cells, pericytes, CNS border-associated macrophages (BAMs) and oligodendrocytes, also play their special role in neurodegeneration.

Pericytes and endothelial cells are critical cellular components of the BBB, which acts as a barrier as well as a bridge for the communication between CNS and periphery. This unique anatomical position gives them especial function in neuroinflammation. In vitro treatment of endothelial cells with Aβ induced expression of multiple inflammatory molecules, including IFN-γ, IL-6, IL-1β, and CD40,^470,471^ increasing the secretion of monocyte chemoattractant, MCP-1, which has been shown to be highly expressed in the cerebral endothelial cells from AD patients.^471^ In addition, in vitro treatment of rat cerebral endothelial cultures with inflammatory mediators (i.e., LPS), cytokines (i.e., TNF-α and IL-1α) or Aβ resulted in the increased CAP37 expression, which plays crucial role in mononuclear cell chemotaxis, adhesion of monocytes to endothelium, and release of oxygen radicals from monocytes.^472^ Transcriptomic analysis suggested that several subpopulations of endothelial cells in AD are associated with immune response which was characterized by increased expression of genes associated with antigen presentation—especially major histocompatibility complex class I (MHC-I) machinery.^473^ These results indicate that stimulating of endothelial cells with Aβ or cytokines activates endothelial cells to be an inflammation initiator to further recruit and activate other immune cells. Activation of brain endothelial cells also impaired integrity of BBB by multiple pathways, which would increase the infiltration of peripheral immune cells. Multiple in vitro experiments revealed that stimulation of endothelial cells with Aβ or proinflammatory cytokines reduced the expression of tight junction proteins like Claudin-5, VE-cadherin and Occludin, thus increased permeability.^474–478^ Mechanically, in vitro studies revealed that stimulation of endothelial cells with proinflammatory cytokines or Aβ can induce the expression iNOS.^479^ In line with these results, iNOS was found to be highly expressed in the endothelial cells of AD patients, indicating an increased generation of NO by endothelium in AD.^480^ Binding of Aβ with endothelial RAGE receptor also induced oxidative stress, as indicated by improved NADPH oxidase (NOX) and increased the generation of ROS (primarily peroxynitrite), which ultimately induced inflammation and impaired tight junctions, leading to loss of BBB integrity^481,482^ and interruption of cerebral blood flow.^483^ In addition, exogenous factors can also affect the integrity of endothelial cells. Serum amyloid A (SAA), an acute phase protein secreted by hepatocytes, was suggested to decrease claudin-5 expression and transendothelial electrical resistance, increase sodium fluorescein permeability of endothelial cells in vitro, indicating a liver-to-brain inflammation axis.^484^ In addition, HIV-1 Tat C was suggested to upregulate miR-101, which led to downregulation of tight junction protein VE-cadherin and impaired permeability.^485^ These two studies also suggest that neurodegenerative diseases, including AD, are probably induced by exogeneous factors.

Generally, leukocytes do not interact with resting endothelial cells. However, the intercellular adhesion molecule-1 (ICAM-1) was shown to be highly expressed in endothelial cells of AD patients.^486^ In vitro stimulating of human brain endothelial cells with Aβ or TNF-α induced the expression of endothelial adhesion molecules, including selectin, ICAM-1, and vascular cell adhesion protein 1 (VCAM-1), which are responsible for the adhesion and transport of leukocyte in to CNS.^487,488^ Recently, C3a/C3aR signaling in endothelial cell was suggested to increase VCAM-1 expression and reduce tight junction expression, ultimately impair barrier integrity.^489,490^ It’s also interesting to find that plasma levels of the soluble VCAM1 (sVCAM1) are highly increased in PD patients compared with age-matched healthy controls. Of note, sVCAM1 levels correlated significantly with disease severity.^491^ In in vitro study, Aβ/RAGE interaction also induced the expression of CCR5 receptor, which was suggested responsible for the migration of MIP-1α (a CCR5 ligand)-expressing T cells (isolated from AD patient) through in vitro BBB model,^492^ and the expression of cationic antibacterial proteins, which enhanced the adhesion of monocytes to endothelial cells and exacerbated inflammatory reaction in endothelial cells.^493^

**Fig. 7:**
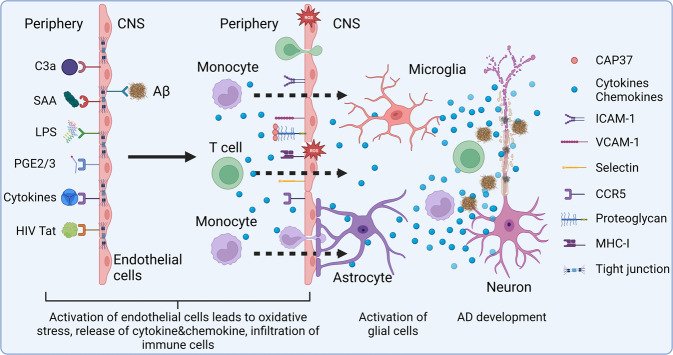
Involvement of endothelial cells in AD. Endogenous stimuli (e.g., Aβ aggregates) or exogenous stimuli (e.g., C3a, SAA, LPS, prostaglandins, cytokines, virus proteins) can bind to its receptors in endothelial cells and induce the activation of endothelial cells. Activated endotheliocyte secrets a low level of inflammatory cytokines and chemokines, increases the expression of adhesion molecules and reduces the expression of tight junction proteins. Activation of endothelial cells also increases the expression of iNOS and NOX, which lead to oxidative stress, amplifying inflammation and damaging BBB. Secretion of cytokines and chemokine recruits peripheral immune cells, activates microglia and astrocytes in the CNS. The impaired permeability of endothelial cells and increased expression of adhesion molecules facilitate the infiltration of peripheral immune cells into the CNS, further augmenting inflammation in the CNS. As a result, stimulation on endothelial cells leads to strong inflammation in the CNS, ultimately induces Aβ and tau aggregation, and neurodegeneration

In summary, although endothelial cells produce relatively lower levels of cytokines compared with astrocytes and microglia because they are located at the interface between the CNS and periphery which makes them continuously exposed to potential toxic in the brain or blood, they play a crucial role to initiate a destructive inflammatory cycle in the CNS. Thus, after being activated by toxic in the brain or blood, endothelial cells release low levels of cytokines which lead to paracrine stimulation of glial cells in the CNS and recruitment of peripheral immune cells, ultimately leading to chronic inflammation and neural toxicity^494^ (Fig. 7).

Pericytes are multiple-functional mural cells embedded in the wall of capillaries and key regulator of BBB integrity. Many neurodegenerative diseases were found to be associated with the loss of pericytes.^495,496^ After being activated by proinflammatory mediators like LPS, TNF-α and Aβ, pericytes can secret multiple cytokines including TNF-α, IL-6 and chemokine (C-X-C motif) ligand 1 (CXCL1), as well as increasing the expression of receptors for inflammatory signals, such as Toll-Like Receptor 4 (TLR4).^497–499^ In addition, pericytes are also suggested to be an important source of pro-inflammatory eicosanoids after being activated by Aβ.^500^ This means that pericytes are not only respond to inflammation but also important modulators of inflammation. In a transgenic mouse carrying human APOE4 (a major genetic risk factor for AD), APOE4 secreted from astrocytes was found to bind with LRP1 on pericytes and induce uncontrollably proinflammatory CypA expression in pericytes, inducing the activation of NF-kB and matrix metalloproteinase 9 (MMP9),^183,501^ which has been suggested to be increased in leptomeningeal vessels of AD patients.^502^ In addition, reduced expression of tight junction proteins was found in AD mouse model with increased MMP9 activities.^183^ These results explain the mechanism of MMP9 in regulating BBB integrity. In multiple animal models, inhibition of CypA-MMP pathway improved BBB integrity, reduced loss of neurons and attenuated behavioral deficits.^501^ In AD patients, BBB damage is associated with loss of pericytes,^503^ while the decrease in pericytes is followed by an increase in activated microglia.^504^ The loss of pericytes is probably caused by increased expression of friend leukemia virus integration 1 (Fli-1) after being activated.^503^ Fli-1 is an important regulator of inflammation and was reported to enhance the proinflammatory cytokine secretion from lung pericytes. Li et al. found that the expression of Fli-1 in pericyte was elevated in the hippocampus of AD patients and in 5xFAD mice. Fli-1 inhibition reduced the apoptosis of pericyte and the secretion of TNF-α and IL-6 in the hippocampus of 5xFAD mice.^499^ This study indicated that Fli-1 expression may affect the apoptosis and activation of pericyte in BBB, leading to microvascular inflammation and BBB dysfunction. But the drawback of this study is that the inhibition of Fli-1 is not specific to pericytes in vivo, further studies are needed to verify the role of Fli-1 in the proinflammatory activation of pericytes. In addition, deficient PDGFRβ signaling in AD also can lead to pericyte loss causing BBB disruption independently of Aβ.^505^ In vitro cultured pericytes were suggested to rapidly clear extracellular Aβ40 via LRP1, while excessive accumulation of Aβ in pericytes over longer periods of time resulted in cell death.^506^ Pericytes dysfunction may also involve in the pathology of ALS. IL-6 is a cytokine with pleiotropic effects, difference in expression level may lead to adverse effects. The proper expression of IL-6 by pericytes was suggested to be important for the integrity of BBB.^507,508^ In an in vitro experiment, Scotter et al. found that dysfunction of TDP-43 in pericytes suppressed the expression of IL-6 by pericytes,^509^ indicating that TDP-43 loss-of-function may contribute to the pathology of ALS by disrupting the function of pericytes. The crosstalk between endothelia and pericytes was also important for regulating neuroinflammation. In pericyte-deficient mouse line Pdgfb^ret/ret^, the expression of VCAM1 and ICAM1 was significantly increased on the brain vasculature, which was accompanied by increased leukocyte infiltration into the brain parenchyma.^510^ Using complex in vitro models, Kozma et al. demonstrated that cerebral endothelial cells and pericytes can mutually activate inflammasome dependent signaling of each other after being activated by stimulus from the brain or peripheral, through which way they closely communicated to exchange the stimulus signals between CNS and peripheral.^511^ Owing to the numerous roles of pericytes in neuroinflammation,^512,513^ they are thought to be an attractive therapeutic target for AD therapy by tuning neuroinflammation. Taking together, pericytes are involved in the clearance of aggregates and play important role in the integrity of BBB. Activation of pericytes induces the expression of proinflammatory cytokines and eicosanoids, and leads to the apoptosis of pericytes through multiple ways. Pericytes activation also increases the expression of MMP9 which enhances the degradation of ECM collagen and tight junction proteins. As a result, pericytes activation increases the permeability of brain vascular, and infiltration of immune cells and exacerbates neurodegeneration.

There are three main types of macrophages in the CNS, including perivascular macrophages (PVMs), meningeal macrophages, and choroid plexus macrophages. All of them are localized at anatomical borders between the CNS and periphery, thus they are named BAMs.^514^ However, recent studies using high-dimensional single-cell analysis revealed that BAMs display regional heterogeneity and can be classified into multiple subtypes with diverse signatures.^515,516^ Importantly, the special anatomical location of BAMs confers them unique function in CNS homeostasis. PVMs were the most studied group among BAMs associated with neurodegeneration. PVMs were suggested to enrich with scavenger receptors, indicating that they may be involved in the clearance of dangers from the cerebral parenchyma.^517,518^ In a mutant APP transgenic mice, depletion of PVMs increased the deposition of amyloid-β around cerebral blood vessels.^519^ In addition, PVMs also participate in Aβ induced neurovascular dysfunction through CD36 mediated oxidative stress, leading the infiltration of immune cells.^520,521^ Single-nucleus RNA sequencing analysis suggested that PVMs from patients with AD displayed abnormity in phagocytosis, endocytosis, and interferon-γ signaling.^522^ However, the mechanism of PVMs in the development of AD is still far from being elucidated.

In recent years, numerous studies have suggested that oligodendrocytes or oligodendrocyte progenitor cells are not only cells affected by inflammation, but also a source of inflammation.^523^ But the story of oligodendrocytes in neuroinflammation is just beginning.

## Immune-directed therapies

### Alzheimer’s disease

Since the landmark report by Schenk et al. in 1999 showing that active vaccination with Aβ attenuated deficits in AD mouse model, researchers in this field have sought to develop both active and passive anti-Aβ immunization therapeutics for AD.^524^ Immunization of AD patients with first-generation Aβ vaccines greatly reduced amyloid aggregation, but did not stop disease progression, even leading to the development of meningoencephalitis.^525^ These results prompt the development of second generation Aβ vaccines that limit the induction of self-reactive T cells. While none of the second generation vaccines that underwent clinical trials has induced meningoencephalitis, no clinical benefit has been reported either.^526^ Numerous trials of Aβ antibodies passive transfer have also been performed. Based on encouraging initial results,^527^ aducanumab, an antibody for Aβ, recently finished two large Phase III clinical trials. A dose- and time-dependent reduction in the pathology of AD was observed in both trials.^528^ Thus, the US FDA has approved aducanumab as the first disease-modifying treatment for AD in 2021.^529^ In January 2023, Lecanemab, the other antibody for Aβ, was also approved by using the US FDA’s accelerated approval pathway as a treatment for early AD.^530^

Considering that tau pathology has better correlation with cognitive impairments than Aβ plaques, tau targeted therapy is thought to be a better choice than Aβ. At present, due to the toxicity or lack of efficacy of other anti-tau therapies, most of the tau targeted therapies undergoing clinical trials are immune-based therapies.^531^ Sigurdsson’s group was the first to report successful tau targeted therapy based on active and passive immunization against tau, which reduced pathological tau levels and attenuated the behavioral phenotypes associated with tauopathy.^532^ These results were later confirmed by other groups. Some studies reported that tau immunization induced toxicity owing to the generation of self-reactive T cells,^533,534^ though this may have been caused by the use of strong adjuvants. The passive transfer of anti-tau antibodies has also been shown to be an effective method, since it was reported that tau can be secreted into the extracellular space,^535^ and some antibodies even can enter neurons.^531^ At present, multiple clinical trials based on tau active and passive immunization are ongoing since the tests in the animal models have yielded promising results.^531^

TREM2 has emerged as key signaling hub in AD, as discussed above. The TREM2 pathway can be targeted by specific antibodies or small molecules that regulate downstream signaling.^211^ AL002, an monoclonal antibody of TREM2, has undergone Phase II clinical trial for the treatment of early AD.^536^ In an AD mouse model, administration of AL002c, a variant of the AL002 antibody, attenuated tau plaques and neurite dystrophy, improved behavior, and reduced microglial inflammation.^537^ Other drugs targeting TREM2, including DNL919 and AL044, are also undergoing clinical trials (clinicaltrials.gov).

Besides activating TREM2 using agonistic antibodies, improving the level of TREM2 is also a promising method for attenuating neurodegenerative disease. The level of TREM2 on the surface of cell membrane is regulated by a disintegrin and metalloproteinases (ADAMs) which constantly cleave full-length TREM2 into sTREM2, regulating the speed of ADAMs mediated shedding is another way to intervene in TREM2 signaling.^538^ A dual-function TREM2 antibody, 4D9, has been developed to reduce the shedding of TREM2 by ADAMs and concomitantly activate pSYK signaling.^538^ The 4D9 antibody improved the survival of macrophages and increased the uptake of myelin debris and Aβ peptides by microglia. Administration of 4D9 in AD mouse model increased the level of TREM2 in microglia and reduced amyloidogenesis,^539^ though no changes to cognitive ability were reported. The researchers are trying to move a human version of this antibody toward clinical trials. Lastly, using antisense oligonucleotides that potently but transiently reduce TREM2 expression at late stages of plaque pathology significantly reduced plaque deposition and microglia association around plaque deposits, indicating a time- and/or dose-dependent intervening the TREM2 signaling for the therapy of AD.^540^

Epidemiological data have suggested that NSAIDs are associated with increased risk for AD, but multiple clinical trials have failed to bear that out.^541^ However, scientists didn’t stop to develop anti-inflammatory drugs for AD therapy, owing to the crucial role of inflammation in neurodegeneration. The combination of ibuprofen and cromolyn, two FDA approved drugs, generated exciting results in the treatment of AD mouse model. Thus, ALZT-OP1, a combination regimen of ibuprofen and cromolyn, has finished Phase 1/2 clinical trials. A Phase III clinical trial is ongoing (clinicaltrials.gov). Minocycline, as an anti-inflammatory tetracycline that crosses the BBB and inhibits proinflammatory microglia, was identified as a high-priority drug for clinical trials in AD. In a transgenic AD mouse model, minocycline prevented Aβ deposition and neuronal death, reduced tau phosphorylation and aggregation, and improved cognitive deficits; however, it failed to delay disease progression in individuals with mild AD in a clinical trial.^542^ In addition, natural formulas with anti-inflammatory activity have been developed to treat AD.^543^ Neurodegenerative diseases involve complex immune regulation, which makes them difficult to treat with mono-target therapy. Nutraceuticals consisting of multiple natural ingredients that can target to multitargets showed antioxidant and anti-inflammatory properties, as well as the ability to disassemble tau oligomers in clinical trial.^544^

As we discussed above, many receptors (i.e., TLR, P2Y6, CSF-1R), signal transducers (i.e., JAK, p38 MAPK, ERK), activators of transcription (i.e., STAT, NF-κB) and inflammatory mediators (i.e., GM-CSF, TNF-α, NO) are involved in neuroinflammation and AD development. Small molecules or monoclonal antibodies targeted to these signaling pathways have also been developed and tested in clinical trials (clinicaltrials.gov). Semaphorin 4D (Sema4D) has been suggested to be upregulated in stressed or damaged neurons and signal through plexin-B1/B2 receptors to activate glial cells and endothelial cells, contributing to the development of neurodegenerative diseases.^545^ In May 2020, pepinemab, a monoclonal antibody to Sema4D has been registered in a Phase I/II clinical trial by Vaccinex for the treatment of AD (clinicaltrials.gov).

As we discussed above, dietary habits, obesity, and diabetes are associated with chronic inflammation, increasing the risk of developing neurodegenerative diseases. Therapies previously developed for treating diabetes are now undergoing preclinical and clinical trials for AD. For example, metformin, as a first-line medication for type 2 diabetes mellitus, has been reported to be a “magic” drug that might benefit various diseases, including AD. In addition, other drugs previously used for diabetes, such as liraglutide, insulin, pioglitazone, and metformin, have shown effectiveness in treating AD, and many are undergoing clinical trials.^546^

Mutations that reduce PGRN levels have been suggested to be risk factors for many neurodegenerative diseases including AD. PGRN overexpression is protective in animal models of AD.^547^ Small molecule like ZAP2006 and monoclonal antibody like AL101 that can enhance the expression of PGRN have been developed and undergoing clinical trials for the treatment of AD (clinicaltrials.gov).

Healthy diets that are low in calories and enriched in ω-3 fatty acids may decrease the risk of developing AD. Epidemiological studies revealed that increased uptake ω-3 of PUFAs reduced the risk of dementia.^548^ In clinical trials, administration of ω-3 PUFAs, the substrate of many SPMs, only benefited the AD patients with mild cognitive impairment, but had no affection on advanced AD patients.^549,550^ In other clinical studies, administration of DHA alone also had no affection on the cognitive impairment of AD patients.^551,552^ These diverse outcomes may be resulted from the variable quality of ω-3 PUFA supplements on the market^553^; larger and more rigorous clinical trials on the effect of ω-3 PUFA in AD treatment are needed in the future. Clinical trials of other immune-modulatory drugs targeted to different inflammatory molecules or cells, including sodium oligomamate, daratumumab, lomecel-B, IBC-Ab002 and protollin, have also made some progresses (clinicaltrials.gov).

### Parkinson’s disease

Immune-mediated therapies were believed to have promise in treating PD, although most of the trials thus far have had disappointing results. The transmission of α-synuclein between different cells has been reported to be a cause of PD progression,^70^ indicating α-synuclein transmission as a new possible target for therapy. Positive vaccination or passive antibody transfer-mediated immune therapy targeted to extracellular tau can reduce the aggregation of α-synuclein and attenuate disease. In 2005, Masliah et al. first reported that immunizing human α-synuclein transgenic mice with purified recombinant human α-synuclein reduced α-synuclein aggregation in neuronal cell bodies and synapses and decreased neurodegeneration.^554^ Since then, numerous studies have tested this strategy using different designs of the vaccine.^555–557^ Three of the vaccines, AFFITOPE PD01A, AFFITOPE PD03A, and UB-312, are ongoing Phase II clinical trials.^49,558^ Multiple monoclonal antibodies targeting different motifs on α-synuclein have also been designed and tested in clinical studies, including ABBV-0805, LU AF82422, Prasinezumab, TAK-341 and UCB7853. NPT200-11, a small molecule inhibitor of α-synuclein aggregation, is also undergoing Phase II clinical trial.^559^

As the common characteristic of all neurodegenerative diseases, inflammation has always been concerned as a target for therapy, although epidemiological studies on the effect of NSAIDs on the progression of PD have yielded inconsistent results. Alternative drugs with anti-inflammatory properties, including dexamethasone, minocycline, naloxone, are going to be or have already been tested in preclinical and clinical trials.^560–562^

Given the vital role of the NLRP3 inflammasome in the pathogenesis of PD, researchers have suggested targeting NLRP3 signaling as a potential therapeutic strategy. Inhibiting the expression of NLRP3 by microRNA can ameliorate α-synuclein aggregation and protect dopaminergic neurons against degeneration in a mouse model of PD.^360^ MCC950, a specific NLRP3 inhibitor, exerted neuroprotective effects and improved behavioral dysfunctions in an MPTP-induced PD mouse model.^563^ These exciting results in animals led them to seek a clinical trial. The other NLRP3 inhibitor, inzomelid, has completed Phase I clinical trial. Another small molecule inhibitor, kaempferol, has also been developed to block NLRP3 signaling.^564^ Targeting caspase-1, a downstream molecule of NLRP3, is another promising method, which was found to decrease the secretion of IL-1β and attenuate the susceptibility of dopaminergic neurons.^358^

TLR signaling plays a significant role in initiating inflammatory responses: it increases the expression of cytokines and oxidative stress molecules in response to PAMPs intruding from outside or DAMPs released from damaged cells. α-synuclein can act as a DAMP to activate microglia through TLRs receptors, thus blocking TLR signaling may be a therapeutic strategy for attenuating PD.^565^ Administration of anti-TLR2 antibody to a PD mouse model decreased the accumulation of α-synuclein, reduced inflammation, and improved behavioral deficits.^566^ CU-CPT22, a small molecule that specifically inhibits TLR1 and TLR2 receptors, reduced activation of the NF-κB pathway and secretion of the proinflammatory cytokines IL-1β and TNF-α in vitro,^567^ as well as ameliorated motor and sensory function in MPTP-induced PD mouse model in vivo.^568^ The other small molecule named NPT520-34 has completed Phase I clinical trial for the treatment of PD. Small molecules, such as kaempferol, farrerol, and schisandrin, were shown to inhibit inflammation through the TLR4 pathway in vitro and in vivo, indicating their potential to be used for PD therapy.^569–571^

Due to the close association between diabetes and PD, drugs previously approved for diabetes therapy have also been tested for PD. GLP-1 is a well-characterized target for diabetes. In a Phase II clinical trial, exenatide, an agonist of the GLP-1 receptor, showed great protective effects in PD patients.^572^ NLY01, a different GLP-1 receptor agonist that can protect dopaminergic neurons from inflammatory responses elicited by glial cells,^573^ has been enrolled recently in a Phase II clinical trial. The GLP-1 analogue semaglutide showed multiple benefits in a MPTP-induced PD mouse model: it reduced the accumulation of α-synuclein, alleviated chronic inflammation, attenuated the peroxidation of lipid, inhibited the mitophagy related signaling and increased the expression of glial cell line-derived neurotrophic factor (GDNF).^574^ A clinical trial testing semaglutide in PD is ongoing. A Phase III clinical trial is also undergoing for exenatide, an analogue of GDP-1, as a potential disease-modifying treatment for PD.^575^ Clinical trials of other drugs including AKST4290, BIIB094, DNL151, DNL201, NE3107 and sargramostim targeting to different immune modulatory pathways have also made some progress (clinicaltrials.gov).

Since α-synuclein derived antigens specific T cells have been validated in PD, many researchers have attempted to attenuate this T cells mediated adaptive immunity as a therapeutic approach to prevent the progression of disease. Some studies have attempted to increase Treg responses.^576^ But all these studies were done in PD animal model. In the future, clinical trials are needed to further verify the effectiveness of these kinds of therapeutic methods.

### Amyotrophic lateral sclerosis

Multiple small molecules with anti-inflammatory activity, including minocycline, masitinib, NP001, and celecoxib, have been tested for the treatment of ALS. Although most have failed, masitinib was found to be effective in the preceding clinical trials, and a Phase III clinical trial testing masitinib in ALS patients is ongoing.^577^ dl-3-n-Butylphthalide (dl-NBP), a synthetic compound based on l-3-n- Butylphthalide that is isolated from seeds of Apium graveolens, has been found to reduce glial cell activation, attenuate motor neuron death, and prolong the survival interval of SOD1^G93A^ mice. A double-blind Phase II clinical trial with oral administration of NBP in ALS patients is ongoing in China.^78^ Other anti-inflammation drugs currently being tested include ibudilast and RNS60.^78^ Recently, edaravone, a free radical scavenger with antioxidant effects, was approved in Japan in 2015 and then in the US in 2017 as a treatment for ALS.^578^

Given the importance of PGRN in ALS development, AL001, a human monoclonal antibody that blocks the sortilin-PGRN interaction to prevent the degradation of PGRN, is currently being investigated in a Phase II study to evaluate its safety, pharmacokinetics, and pharmacodynamics in ALS-C9orf72.^547^ As other neurodegenerative disease, drugs targeted to some key molecules involved in inflammatory signaling pathways, including TLR2, IL-1R, IL-6R, JAK and receptor-interacting serine/threonine-protein kinase 1 (RIPK1), have also been developed. Clinical trials for these drugs are under the way (clinicaltrials.gov). Absence of Sigma-1 receptor (Sigma-1R) induces mitochondrial dysfunction which is an important mechanism underlying ALS development. Based on this theory, Sigma-1R targeted therapy has been thought to be a promising method. Pridopidine, a Sigma-1R agonist, has been enrolled in a Phase III clinical trial for treatment of ALS.^579^ Fingolimod is an immunomodulating medication which can reduce infiltration of lymphocytes into the CNS. It was mostly used for treating multiple sclerosis. Recently, fingolimod was found to be protective in ALS mice model,^580^ a Phase II clinical trial is ongoing (Table 1).

**Table 1:** Immuno-therapeutic strategies in clinical trials for neurodegenerative diseases

Disease	Targets	Name of drug	Phase of clinical trial
AD	Aβ targeted	Trontinemab (RG6102)	Phase I
		Remternetug (LY3372993)	Phase III
		PRX012	Phase I
		MEDI1814	Phase I
		GSK933776	Phase I
		ALZ-101	Phase I
		ACU193	Phase I
		ABBV-916	Phase II
		ACI-24	Phase II
		Donanemab (LY3002813)	Phase III
		AN-1792 (QS-21)	Phase II
		Amilomotide (CAD106)	Phase III
		Vanuitde cridificar (ACC-001)	Phase II
		ABvac40	Phase II
		Lu AF20513	Phase I
		UB-311	Phase II
		Bapineuzumab (AAB-001)	Phase III
		Solanezumab (LY2062430)	Phase III
		Crenezumab (RG7412)	Phase III
		Gantenerumab (RO4909832)	Phase III
		Aducanumab (BIIB037)	Approved
		Lecanemab (BAN2401)	Approved
		Ponezumab (PF-04360365)	Phase II
		AV-1959D	Phase I
		Affitope AD02	Phase I
	Tau targeted	AADvac1	Phase II
		ACI-35 (VAC20121)	Phase II
		Gosuranemab (BIIB092)	Phase II
		Tilavonemab (ABBV-8E12)	Phase II
		Semorinemab (RO7105705)	Phase I
		Zagotenemab (LY3303560)	Phase I
		JNJ-63733657	Phase I
		Bepranemab (UCB0107)	Phase II
		BIIB076	Phase I
		APNmab005 (RAA7)	Phase I
		E2814	Phase II
		Lu AF87908	Phase I
		MK-2214	Phase I
		PNT001	Phase I
		PRX005	Phase I
	TREM2 targeted	AL002c	Phase II
		DNL919	Phase I
		AL044	Phase I
	TNF-α signaling	Etanercept	Phase II
		Pegipanermin (XPro1595)	Phase II
		Lenalidomide	Phase II
	NO signaling	CY6463	Phase II
	TLR4 signaling	TB006	Phase I
	CSF-1R signaling	Edicotinib	Phase I
	P2Y6 signaling	GC021109	Phase I
	NLRP3 inflammasome	Inzomelid	Phase I
	GM-CSF signaling	Sargramostim	Phase II
	JAK inhibitor	Baricitinib	Phase II
	p38MAPK	MW150	Phase II
		Neflampimod	Phase II
	ERK, NF-κB, TNF-α	NE3107 (HE3286)	Phase III
	Sema4D signaling	Pepinemab	Phase II
	Diabete drugs	Insulin	Phase II
		Liraglutide	Phase I
		Metformin	Phase III
		Pioglitazone	Phase II
	NSAIDs	ALZT-OP1	Phase III
	Multiple targets	Nutraceutical compound BrainUp-10	Phase II
	Progranulin	AL101	Phase I
		AZP2006	Phase I
	Regulating gut microgiota	Sodium oligomamate	Phase III
	CD38+CD8+ T cell depletion	Daratumumab	Phase II
	MSC based inflammatory modulation	Lomecel-B	Phase II
	PD-L1 (increase imunomodulatory macrophages)	IBC-Ab002	Phase I
	Inflammation resolution	DHA	Phase IV
	Peripheral monocytes activation	Protollin	Phase I
PD	α-synuclein targeted	ABBV-0805 (BAN0805)	Phase I
		Affitope PD01A (ACI-7104)	Phase II
		Lu AF82422	Phase I
		Prasinezumab (PRX002)	Phase II
		TAK-341 (MEDI1341)	Phase I
		UB-312	Phase II
		UCB7853	Phase I
		UCB0599 (NPT200-11)	Phase II
	Anti-inflammation	Dexamethasone	Phase II
		Minocycline	Phase II
		Naloxone	Phase III
	NLRP3 inflammasome	Inzomelid	Phase I
	TLR2 signaling	NPT520-34	Phase I
	Diabete drugs	NLY01	Phase II
		Exenatide	Phase III
	CCR3 signaling	AKST4290	Phase II
	LRRK2	BIIB094	Phase I
		DNL151	Phase III
		DNL201	Phase I
	ERK, NF-κB, TNF-α	NE3107 (HE3286)	Phase II
	GM-CSF signaling	Sargramostim	Phase I
ALS	Anti-inflammation	Masitinib	Phase III
		Ibudilast	Phase II
		RNS60	Phase I
		NBP	Phase II
	Anti-oxidant	Edaravone	Approved
	JAK signaling	Baricitinib (NCB28050)	Phase I
	Progranulin	Latozinemab (AL001)	Phase II
	TLR2 signaling	NPT520-34	Phase I
	RIPK1 signaling	DNL747 (SAR443060)	Phase I
		DNL788 (SAR443820)	Phase II
	IL-1R	Anakinra	Phase II
	IL-6R	Tocilizumab	Phase II
	Sigma-1R (Microchondrial function)	Pridopidine (ACR16)	Phase III
	Reduce circulating lyphocytes	Fingolimod	Phase II

The drugs with multiple targets or unknown targets are classified into anti-inflammation drugs. The drugs that have been discontinued are not included

*AD* Alzheimer’s disease, *PD* Parkinson’s disease, *ALS* Amyotrophic lateral sclerosis, *Aβ* Amyloid β-protein, *TREM2* Triggering Receptor Expressed On Myeloid Cells 2, *NO* Nitric oxide, *TNF-α* Tumor necrosis factor alpha, *TLR4* Toll Like Receptor 4, *CSF-1R* Receptor of the colony-stimulating factor-1, *P2Y6* P2Y purinoceptor 6, *NLRP3* NLR Family Pyrin Domain Containing 3, *GM-CSF* Granulocyte-macrophage colony-stimulating factor, *JAK* Janus kinase, *p38MAPK* p38 mitogen-activated protein kinases, *ERK* Extracellular signal-regulated Kinase, *Sema4D* Semaphorin 4D, *NSAIDs* Non-steroidal anti-inflammatory drugs, *MSC* Mesenchymal stem cells, *PD-L1* Programmed death-ligand 1, *TLR2* Toll Like Receptor 2, *CCR3* C-C Motif Chemokine Receptor 3, *LRRK2* Leucine-rich repeat kinase 2, *NF-κB* Nuclear factor kappa-light-chain-enhancer of activated B cells, *RIPK1* Receptor-interacting serine/threonine-protein kinase 1, *IL-1R* Interleukin-1 receptor, *IL-6R* Interleukin-6 receptor, *Sigma-1R* Sigma 1 receptor, *DHA* Docosahexaenoic acid, *dl-NBP* Dl-3n-butylphthalide

## Conclusions

Pathological protein aggregation was traditional thought as the main initiator of neurodegeneration. Based on this hypothesis, most immunotherapies for neurodegenerative diseases are harnessing the immune system to clear pathological protein aggregates in the past few decades. Clinical trials showed that these treatments appeared to reduce aggregates, but failed to stop disease progression. It has been suggested that neuron damage happened much earlier than the appearance of protein aggregates, so clearance of aggregates might be unable to reverse the neuron impairments. This may partially explain the failure of aggregates clearing therapeutic strategy. However, there should be other factors that cause the continuous worsen of disease after the clearance of aggregates. Dysregulation of the immune response in the CNS and peripheral emerging to be a major concern.

In recent years, growing evidence demonstrates that inflammation not only plays crucial role in promoting the progression of neurodegenerative diseases, but also be an important initiator. For example, inflammation was found to appear much earlier than protein aggregation; constitutive active STING expression is sufficient to induce PD pathology in mouse model. However, anti-inflammatory therapy failed to delay disease progression in clinical trial. This may reflect the complex role of inflammatory signaling in neurodegeneration as we discussed above. Most of the inflammatory signaling involved in neurodegeneration both display beneficial and detrimental roles. In addition, proinflammatory response is important for proper resolving of inflammation. A signaling beneficial in one disease stage might be detrimental in the other stage. These data indicate that timing, cell specificity and target molecules selection in each signaling should be carefully determined to reduce the detrimental role while keep the beneficial role of inflammatory response, which is crucial for successful usage of inflammation targeted therapy. Many therapies specifically targeted to different inflammatory signaling are ongoing clinical trials, probably providing effective therapeutic methods for patients with neurodegenerative diseases in the future. A more complete understanding of the underlying cellular and molecular mechanisms of inflammation in neurodegeneration will be crucial to successfully target inflammatory signals for the treatment of neurodegenerative diseases.
